# Supplementary material for: Enhancing hydrovoltaic power generation through coupled heat and light-driven surface charge dynamics
Source: Nat Commun. 2026 Jan 9;17:1541. doi: 10.1038/s41467-025-68261-8 (PMC12894874; doi:10.1038/s41467-025-68261-8)
Supplement: Supplementary file 1 — Supplementary Information File [file 41467_2025_68261_MOESM1_ESM.pdf]

Supporting Information of the manuscript: Enhancing Hydrovoltaic Power Generation through Coupled Heat and Light-Driven Surface Charge Dynamics

Tarique Anwar[1] and Giulia Tagliabue[1*]

[1]Laboratory of Nanoscience for Energy Technologies (LNET), STI, École Polytechnique Fédérale de Lausanne (EPFL), Lausanne 1015, Switzerland

[*]To whom correspondence should be addressed: E-mail: giulia.tagliabue@epfl.ch

S1: Scanning Transmission Electron Microscopy (STEM)Image of the Silicon Nanopillar

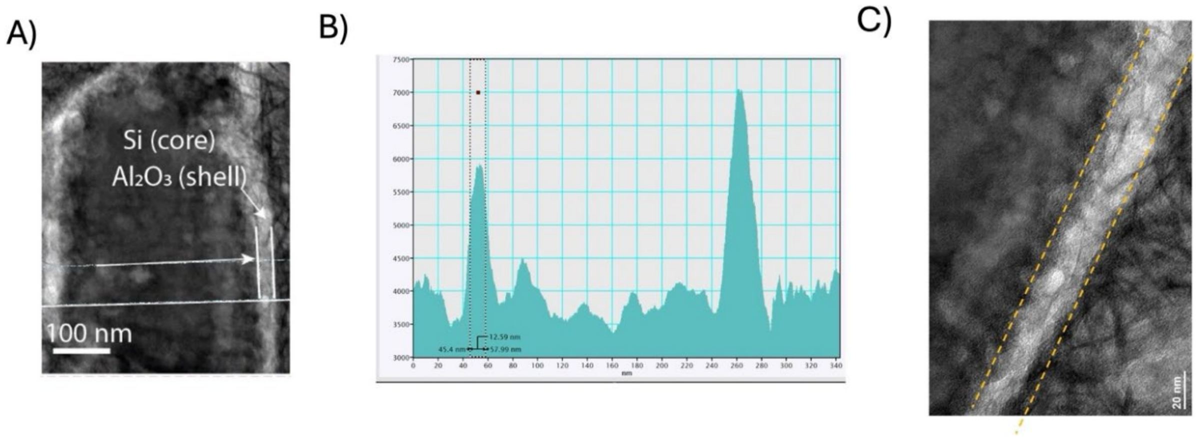

Figure S1: Structural characterization of an individual core–shell silicon–dielectric nanopillar. A) *Scanning Transmission Electron Microscopy (STEM) image of a representative single silicon nanopillar conformally coated with an Al_2O_3 dielectric shell. The core–shell architecture is clearly resolved, with a uniform, continuous shell surrounding the silicon core. Scale bar: 100 nm.* ***B)*** *Corresponding line intensity profile extracted across the nanopillar diameter, used to quantify the thickness of the Al_2O_3 shell. The analysis indicates an average shell thickness of approximately 10 nm, consistent with conformal coating.* ***C)*** *High magnification STEM image of the nanopillar surface, highlighting the presence and uniformity of the oxide layer at the silicon–dielectric interface. Scale bar: 20 nm.*

S2: Surface charge dependence on temperature

In our system, the observed linear relationship between temperature and surface charge arises from the thermally driven shift in the equilibrium of surface ionization reactions. Specifically, temperature increases promote the dissociation of surface hydroxyl groups (e.g., ≡TiOH ⇌ ≡TiO^- + H^+), thereby increasing the density of surface charges.

$$K_a = \frac{[\mathrm{TiO^-}][\mathrm{H^+}]}{[\mathrm{TiOH}]} = \frac{\sigma[\mathrm{H^+}]}{(\Gamma - \sigma)} \quad (S1)$$

The expression for surface charge is obtained in terms of surface proton concentration as show in eq. 1 of the manuscript. To explicitly relate the dependence of surface potential, the equation can be modified as:

$$\sigma = \frac{-e\,\Gamma}{1 + \frac{10^{-pH} e^{-\frac{e(V_{ox-el} - V_{el})}{k_B T}}}{K_a(T)}} \quad (S2)$$

Where, V_{ox-el} is the potential on the oxide—electrolyte interface (**Figure 1e**), V_{el} is the bulk potential of the electrolyte, Γ is the surface site density, K_a is the temperature-dependent equilibrium constant.

The above equation is a more general representation of equation 1 in the manuscript. The relationship between the surface charge and the potential gradient at the interface is well-defined by Eq. S2, which links the surface potential, $V_{ox\text{-}el}$, to the surface charge. It highlights how heat and light are coupled in a highly non-linear manner through the boundary conditions; specifically, illumination and temperature modulate the surface charge through the generation of surface photovoltage (i.e., by changing $V_{ox\text{-}el}$, which depends logarithmically on the light intensity, Eq. 3) and a change in the dissociation constant (K_a, which depends on temperature in a non-linear manner, Eq. S4), respectively.

Furthermore, we note that the temperature dependence of the ionization equilibrium is crucial to our analysis of thermal effects. In our study, we relate the temperature-dependent equilibrium constant *K(T)* to the thermodynamic parameter-enthalpy (ΔH)-via the Van't Hoff equation.

$$lnK_1 - lnK_2 = -\left[\frac{\Delta H}{RT_1} - \frac{\Delta H}{RT_2}\right] \quad (S3)$$

Equivalently, we can write the above equation in the form:

$$K = K_0 \ \exp\left[-\frac{\Delta H}{R}\left(\frac{1}{T}-\frac{1}{T_0}\right)\right] \qquad (S4)$$

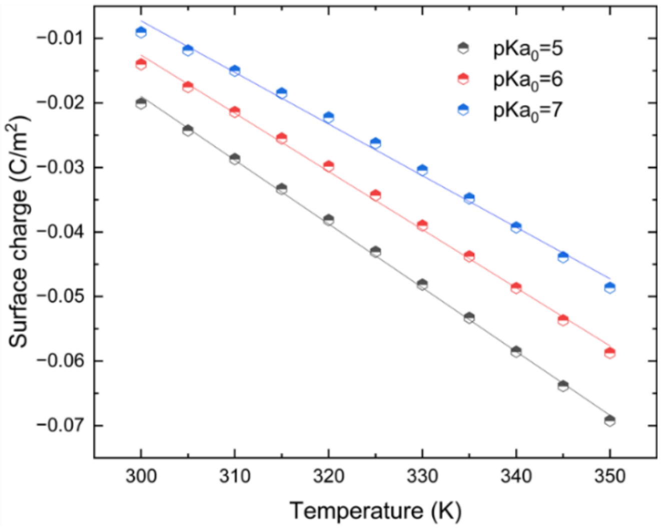

Figure S2: Temperature dependence of surface charge for different surface equilibrium constants. *Variation of the surface charge density as a function of temperature for different values of the surface equilibrium constant, pKa0, evaluated at an initial temperature T_0=300K. The surface charge was calculated using the COMSOL model described in Section S30. For these simulations, a constant enthalpy change, ΔH, was used, as specified in Eq. S3. The results show that variations in the initial value of ΔH do not significantly affect the slope of the surface charge–temperature dependence; instead, they produce an approximately parallel shift of the curves, indicating a systematic offset rather than a change in temperature sensitivity.*

S3: Photoelectrochemical test of the device

We conducted cyclic voltammetry (CV) measurements under 1 sun (1 kW/m^2) illumination across the 0–1 V range at 50 mV/s scan rate. The CV curves show no peaks and onset potentials indicative of faradaic processes, such as water splitting or redox reactions, confirming that no faradaic electrochemical transformations are occurring in the system.

Instead, the observed behavior is consistent with a capacitive charging mechanism, driven by interfacial charge accumulation under illumination. This is further reinforced by the shape of the CV curves (as shown in **Figures S3 and S4**), which exhibit rectangular profiles typical of non-faradaic, capacitive systems.

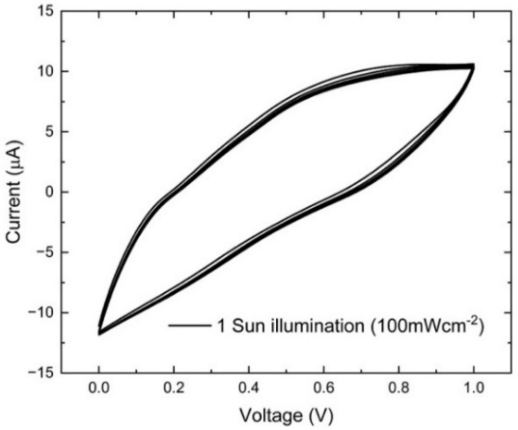

***Figure S3**: **Photoelectrochemical characterization of the SiNP electrode.** Current-Voltage curves under AM 1.5 G illumination of the SiNP electrode in 1 mM KCl.*

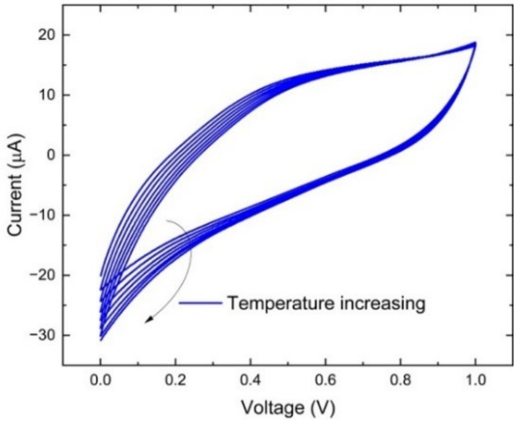

***Figure S4**: **Temperature-dependent electrical characterization of the SiNP electrode.** Current-Voltage curves of the SiNP electrode under increasing temperature in 1 mM KCl. IN this case, illumination was not active. The slight non-linearity in the curve is attributed to continuous changes in temperature.*

S4: Fabrication of core-shell Silicon nanopillars array

Metal-assisted chemical etching (MACE) of crystalline silicon combined with nanosphere lithography was used to fabricate a cm-scale array of SiNPs[1,2]. It involves the self-assembly of polystyrene nanospheres at the water-air interface. Then, the non-closed-packed assembly of PS nanospheres was compressed to a pressure of approximately 25-30(N/m^2) using the Langmuir-Blodgett system, which resulted in a homogeneous closed-packed hexagonal lattice of PS nanospheres[3]. The closed-packed monolayer was transferred to Piranha-cleaned Silicon (P-type 0.1-0.5 Ω.cm, and N-type <0.005 and 1-20 Ω.cm) substrates diced into 2cm X 2cm chips. Plasma etching was used to reduce the diameter of the PS nanospheres with an initial diameter of d =

400*nm*. After the gold-sputtering deposition of a thickness of 20nm and lift-off, a gold nanomesh is formed and used as an etching mask for MACE. Before gold sputtering, 2-3 nm of Ti is sputtered as an adhesion layer. This forms a stable contact between gold and the substrate to avoid delamination during MACE. The liftoff was done by putting the substrate in Toluene and ultrasonicating it at moderate power for 3-5 minutes at room temperature. Finally, MACE was performed by placing the substrate in an aqueous HF/H_2O_2 solution with a volumetric percentage of HF and H_2O_2 as 10% and 2%, respectively. The diameter of the NPs is controlled by changing the time of plasma trimming of the polystyrene nanosphere, while the length of the NPs is controlled by changing the MACE time. The fabricated SiNPs were then surrounded by a shell of dielectric material such as Al_2O_3 and TiO_2 using atomic layer deposition, using trimethylaluminum (TMAl) and titanium tetrachloride ($TiCl_4$) as aluminum and titanium precursors, respectively. The obtained sample was then treated with oxygen plasma (60 sec, 1000 W) to improve the hydrophilicity.

S5: Evaporation rate measured with different electrode sizes

The observed enhancement in evaporation rate arises not from simple surface coverage but from the *physicochemical enhancement* due to the liquid meniscus along the electrode.

Meniscus-Enhanced Flux: When an electrode is inserted, a thin liquid film (meniscus) forms along its entire *vertical length* ($L_{3\Phi}$). Evaporation from this film domain—denoted E_{film} can significantly exceed the bulk evaporation flux, as shown in prior studies[4].

Physicochemical Effects: Beyond geometry, the curved meniscus region concentrates thermal and mass-transfer gradients: i) Thinner films exhibit lower thermal resistance and higher local temperature at the liquid–air interface, boosting evaporation. ii) Steeper vapor-pressure gradients exist near the three-phase contact line, further accelerating local mass flux.

The analytical formulation for the evaporation rates from the bulk and the meniscus region:

$$\frac{dm_{film}}{dt} = \pi D \int_0^{L_{3\phi}} \rho E_{film}(y)\,dy \qquad (S5)$$

$$\frac{dm_{bulk}}{dt} = \rho E_{bulk} A \qquad (S6)$$

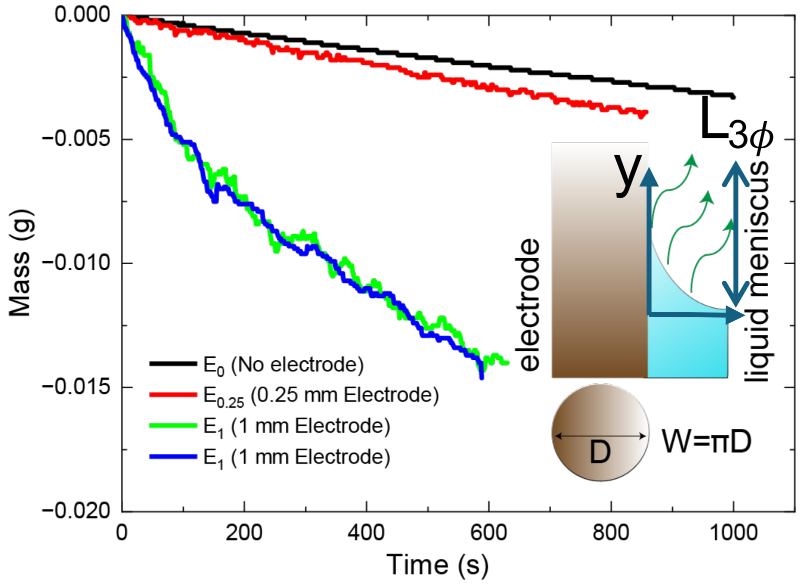

Figure S5**: **Measured evaporative flux of water in different conditions. *The change in mass due to evaporation in an ambient environment was recorded using a microbalance. The Hydrovoltaic cell was placed in the microbalance, then the mass change was monitored in different conditions. I) No electrode was placed in the cell (black curve), ii) 0.25 mm diameter (W=0.8 mm) electrode (red curve), iii) 1 mm diameter (W=3.2 mm) electrode (blue and green curves). The inset shows the meniscus region with perimeter W and length* $L_{3\phi}$.

S6: Hydrovoltaic cell schematic and 3D design

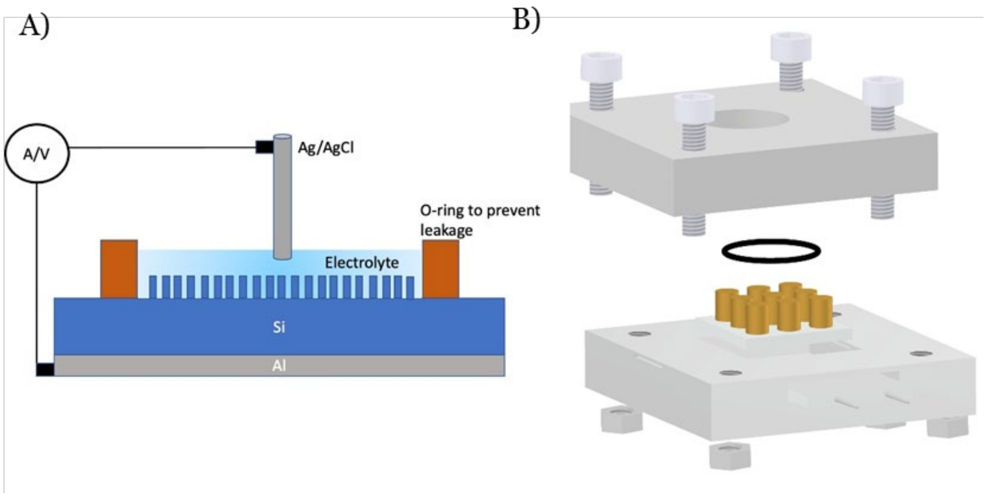

Figure S6: Measurement setup and hydrovoltaic cell design. A) *Schematic of the cross-sectional view of the measurement set-up.* ***B)*** *Three-dimensional rendering of the hydrovoltaic cell highlighting the active central region (~1 cm²) that is selectively wetted with the electrolyte solution, while the surrounding areas of the device remain completely dry. The compression cell is fabricated from VeroClear, a transparent material with properties similar to poly (methyl methacrylate) (PMMA). An O-ring is compressed using four corner screws to ensure a tight seal and to prevent electrolyte leakage beyond the defined active area (~1 cm²).*

S7: Electrical measurements using graphite-electrode

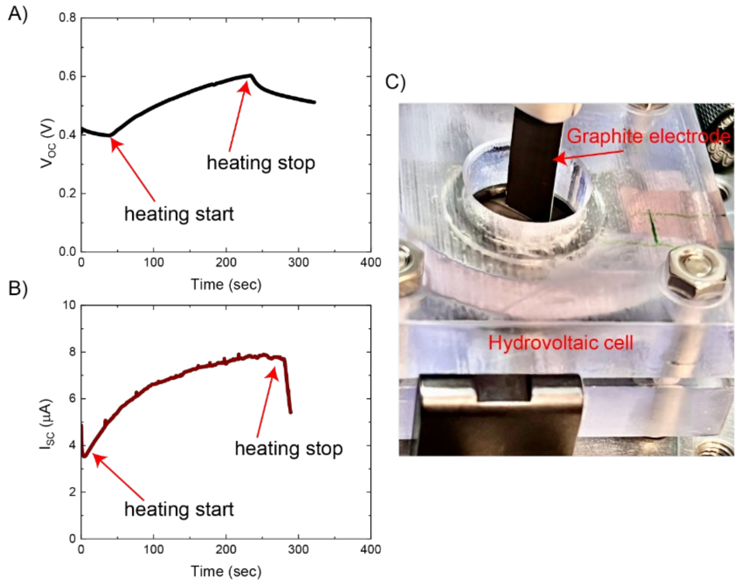

***Figure S7: Electrical measurements using a graphite electrode at ambient, heating, and cooling phases**. **A)** Open circuit voltage, **B)** Short circuit current, **C)** Photo of the Hydrovoltaic cell with the graphite electrode mounted.*

S8: Geometrical asymmetry in the bottom silicon electrode

The measured electrical potential difference is a direct consequence of the formation of the electric double layer. However, the voltage drop is due to a longitudinal imbalance in ionic chemical potential along the length of the silicon nanopillar structures. This imbalance results from asymmetries in the surface charge distribution between the top and bottom of the nanostructure (Figure S8 B).

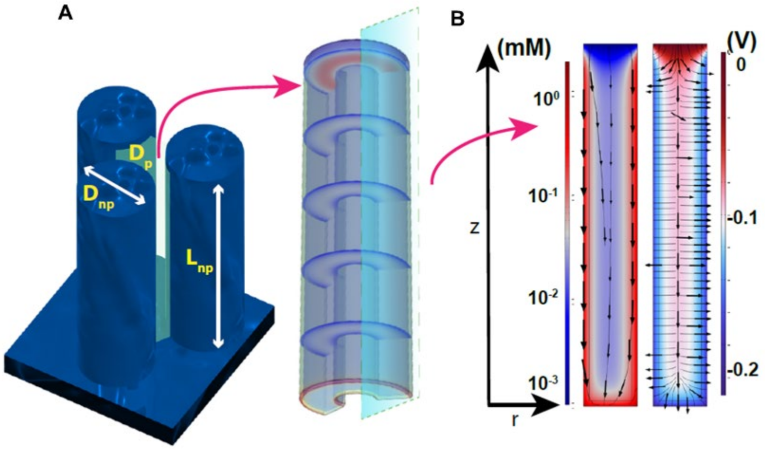

Figure S8: Electrical potential distribution. A) *(Left) Three-dimensional schematic of the triangular unit cell of the hexagonally arranged NPs showing the geometrical parameters of the nanostructures (pillar diameter, D_{np}; pillar length, L_{np}; and mean pore diameter, D_p). (Right) Annular cylindrical nanopore geometry was used for simulations, including the calculated electrical potential distribution.* ***B)*** *Vertical cut plane of the simulated cylindrical nanopore in (A) showing the counterion concentration distribution, with ion flux (left), and the electrical potential distribution, with electric field lines (right). The bulk ionic concentration is 10 μM KCl in the simulation results presented here. Reproduced[2]: Anwar et.al, Device 2, 100287 (2024).*

S9: Voltage-temperature lines obtained using COMSOL Multiphysics model

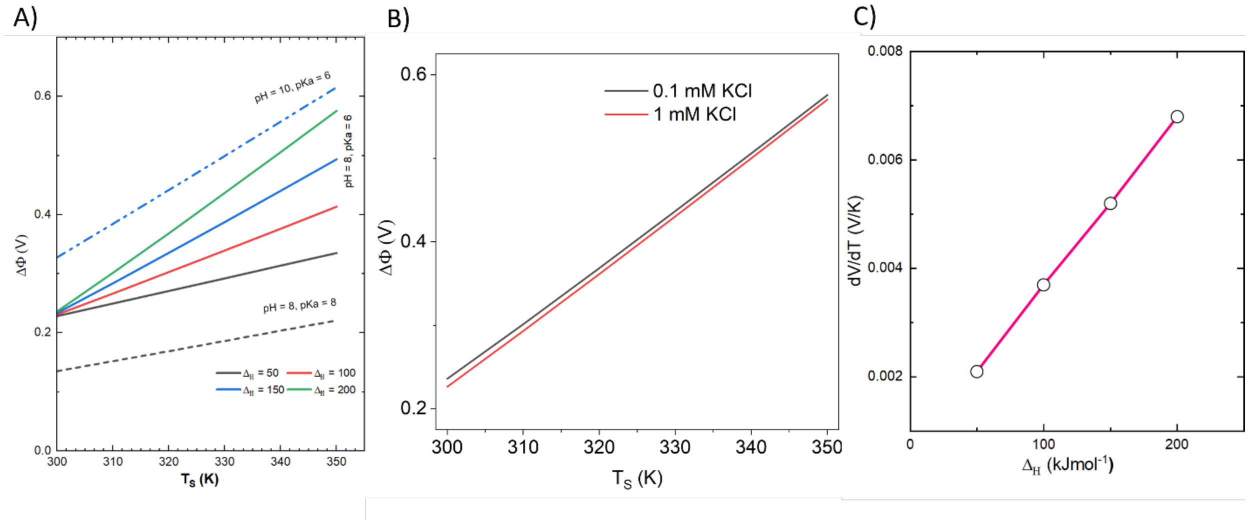

Figure S9: The slope of voltage-temperature lines for various conditions. A) *Chemical potential difference as a function of temperature for various conditions of pH, pKa, and Δ_H. The slope of voltage temperature depends mainly on the oxide's surface chemical characteristic, Δ_H, which is the enthalpy of dissociation of the surface groups[5].* ***B)*** *Chemical potential difference as a function of temperature for 0.1 mM and 1 mM KCl, and pH=8, pka0=6, Δ_H =100.* ***C)*** *The slope dV/dT, plotted as a function of Δ_H, shows a linear dependence.*

S10: Thermal measurements and analysis of system parameters

Heating Configuration:

The Peltier heater was not placed in the liquid but was mounted beneath the SiNPs electrode, in direct contact with the substrate. This configuration ensured that heating was localized and directed upward toward the solid–liquid interface without direct immersion of the heater in the electrolyte.

I: Temperature Monitoring and Time Constants:

We monitored the temperature evolution in real time using an infrared (IR) thermal camera positioned above the system. We have now included the full temperature–time traces for both heating and cooling cycles (**Figure S10-I**) and extracted the corresponding time constants using exponential fits. These reveal the system's characteristic thermal response and are discussed in the revised manuscript.

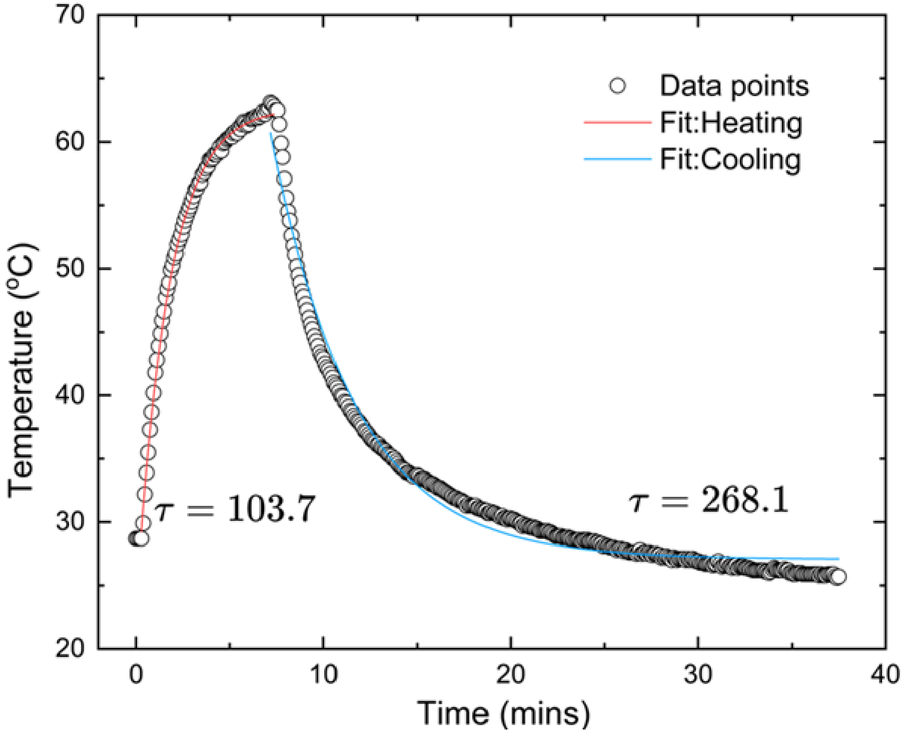

Figure S10-I**: **Temperature measurement of the system. *Measured temperature-time trace during heating and cooling phases. The red and blue curves are the fit to the data points. The distinct values of the heating (103.7 s) and cooling time (268.1 s) constants were obtained, as shown in the figure.*

II: Convective Heat Transfer Role:

We performed a thermal circuit analysis of the system to evaluate the contribution of convective heat losses to the environment. The analysis includes the convective heat transfer coefficient (h) based on natural convection from the top surface of the liquid and the electrode. This coefficient plays a crucial role in the cooling dynamics and temperature stabilization, as it governs the rate of heat dissipation into the surrounding air.

We developed a robust COMSOL model and critically assessed the heat transfer coefficients, h_1, from the bulk water and h_2 from the top electrode, both of which were exposed to ambient air. The temperature of the bottom Silicon was fixed at 70°C, while the ambient temperature was set at 25°C. From our precise measurements, as illustrated in Figure 2B and SI 11, we determined that the temperature difference between the top part of the electrode and the bottom of the Silicon was approximately 7 K at 70°C. Subsequently, we conducted a thorough parametric sweep across various values for h_1 and h_2, ultimately identifying the heat transfer coefficients that yielded a temperature difference closely approximating 7 K. This analysis provided us with estimated values of h1 at 1 W/m²K and h2 at 100 W/m²K. Utilizing these heat transfer coefficient values, we successfully calculated the heat flux to be ~350 W/m²K.

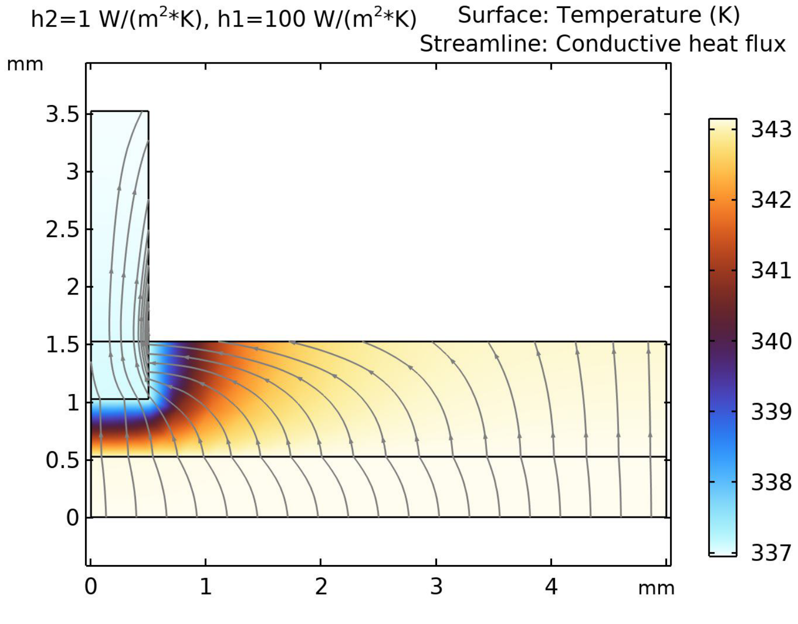

***Figure S10-II**: **COMSOL model for accurately estimating heat flux**. We employed a 2D axis-symmetric approach, incorporating a bottom silicon layer with a thickness of 0.5 mm, an intermediate electrolyte layer measuring 1 mm, and a top silver electrode that has a diameter of 1 mm and a length of 5 mm. The bottom surface was maintained at a fixed temperature, with heat transfer coefficients h_1 and h_2 applied on the solid and liquid sides, respectively.*

Using the above value of heat flux, we estimate the efficiency of the system with respect to Carnot's efficiency:

$$\eta_c = \frac{P_{out}}{P_{in}}\frac{T_H}{\Delta T} = \frac{0.25}{350}\frac{343}{7} = \mathbf{3.5}\ \%$$

III: Thermal Conductivity of Dielectric Oxides:

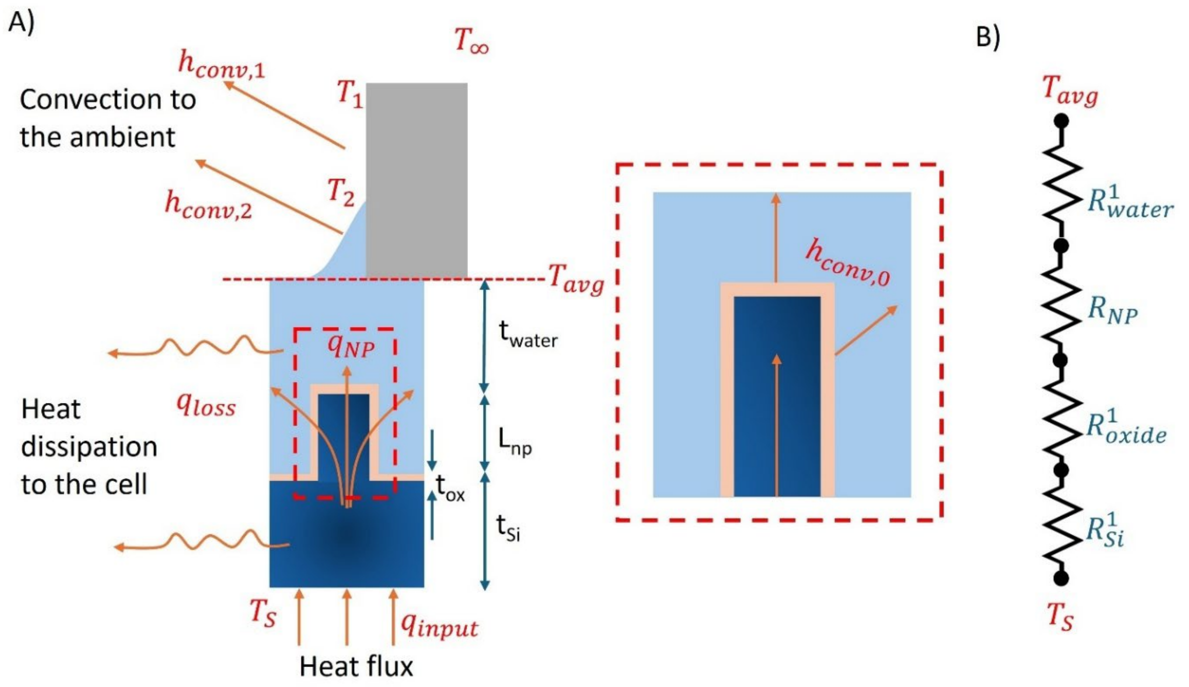

Figure S10-III*: Thermal transport of the system. *A) *The heat flux input on the bottom side of the silicon, alongside the convective heat losses h_1 and h_2 from the liquid and solid sides of the top surface. Furthermore, heat loss into the hydrovoltaic cell, denoted as q_{loss}, is accounted for. The relevant temperatures—T_1, T_2, T_∞, and T_S—represent the temperatures of the top surfaces, ambient conditions, and the bottom silicon. The inset shows the heat transfer in the pin-fin configuration, with the convective heat transfer coefficient h0 as the boundary condition.* ***B)*** *An equivalent thermal circuit with the appropriate thermal resistances is established to ensure accurate analysis. T_{avg} represents the average temperature of the top surface.*

The total thermal resistance is primarily dominated by the bulk silicon and the liquid (which has a larger thickness and a much lower thermal conductivity). Consequently, the overall thermal response is *minimally affected by the oxide's intrinsic thermal conductivity*. Based on one-dimensional heat transfer analysis, we can use the cylindrical pin-fin configuration to estimate the heat transfer coefficients.

Firstly, we can write the equation for heat flux balances, in which we consider the convective heat transfer coefficients on the top surface of the solid (h_1) and liquid (h_2) sides.

$$Q_{input} = Q_{loss} + h_1^{conv} A_1 (T_1 - T_\infty) + h_2^{conv} A_2 (T_2 - T_\infty) \qquad (S7)$$

There are negligible losses from the bottom surface of the silicon; we can write:

$$Q_{NP} = h_1^{conv} A_1 (T_1 - T_\infty) + h_2^{conv} A_2 (T_2 - T_\infty) + Q_{loss} \qquad (S8)$$

Based on the convective heat transfer conditions as the boundary conditions for the silicon nanopillar fin, with base temperature T_S and ambient temperature T_0, we can write:

$$Q_{NP} = \frac{\pi D}{2} \sqrt{h_0 k_{eff} D_{NP}}\, (T_S - T_0)\, tanh(m L_{NP}) \qquad (S9)$$

$$where, \qquad m = \sqrt{\frac{4h_0}{k_{Si} D_{NP} \left(1 + \frac{4 k_{ox} t_{ox}}{k_{Si} D_{NP}}\right)}}$$

Since the shell is thin, we can approximate the effective thermal conductivity of the fin using a parallel resistance model (as heat flows axially through both materials):

$$k_{eff} = k_{Si} \left(1 + \frac{4 k_{ox} t_{ox}}{k_{Si} D_{NP}}\right) \qquad (S10)$$

We can estimate the thermal resistance for the above configuration as follows:

$$R_{NP} = \frac{2}{\pi D_{NP} \sqrt{h_0 k_{eff} D_{NP}}\, \tanh(m L_{NP})} \qquad (S11)$$

$$For\ t_{ox} \approx 10\ nm, \qquad D_{NP} = 400\ nm, \qquad k_{Si} = 50\ \frac{W}{mK}\ and\ \ k_{ox} = 1 - 50\ \frac{W}{mK}$$

$$\frac{4 k_{ox} t_{ox}}{k_{Si} D_{NP}} \ll 1$$

$$k_{eff} \approx k_{Si}, and\ m \approx \sqrt{\frac{4h_0}{k_{Si} D_{NP}}}$$

Therefore, the thermal conductivity of the oxide layer does not significantly affect overall heat transfer coefficients due to its minimal thickness compared to the diameter of the nanopillars.

S11: Analysis of the temperature measurements

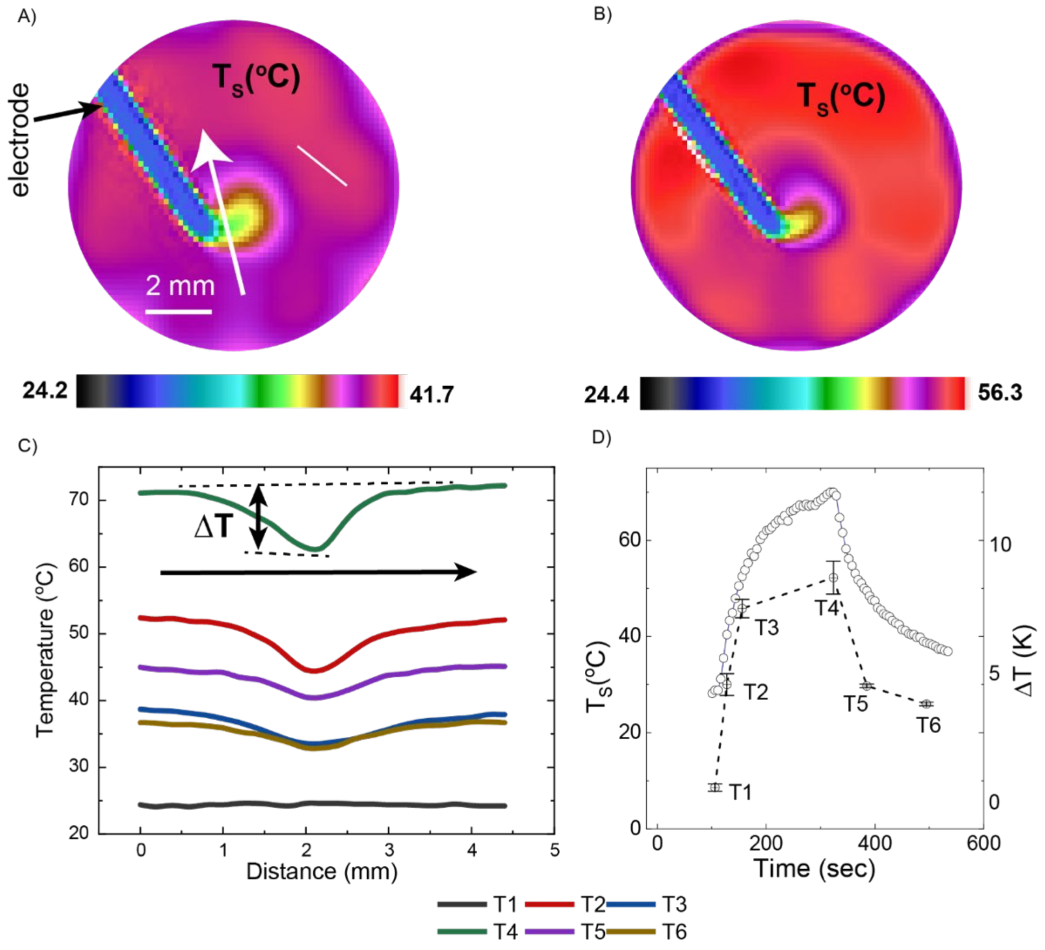

***Figure S11: Temperature maps obtained using an IR camera**. **A)** Temperature map at maximum T_S=41.7°C, **B)** at maximum T_S=56.3°C. **C)** Temperature profile along the indicated white arrow in panel A. **D)** Obtained surface temperature T_S and temperature difference ΔT for the heating and cooling phase.*

S12: Fitting of voltage-temperature curves

We used the measured voltage-temperature curves to obtain the equivalent electrical circuit parameters in **S13.** $V_{OC}^{ij}(T)$ can be fitted using a quadratic temperature dependence, where i stands for different materials, and j is for electrolyte concentrations. The parameters are given in the table below.

$$V_{OC}^{ij}(T) = A_{ij}T^2 + B_{ij}T + C_{ij}$$

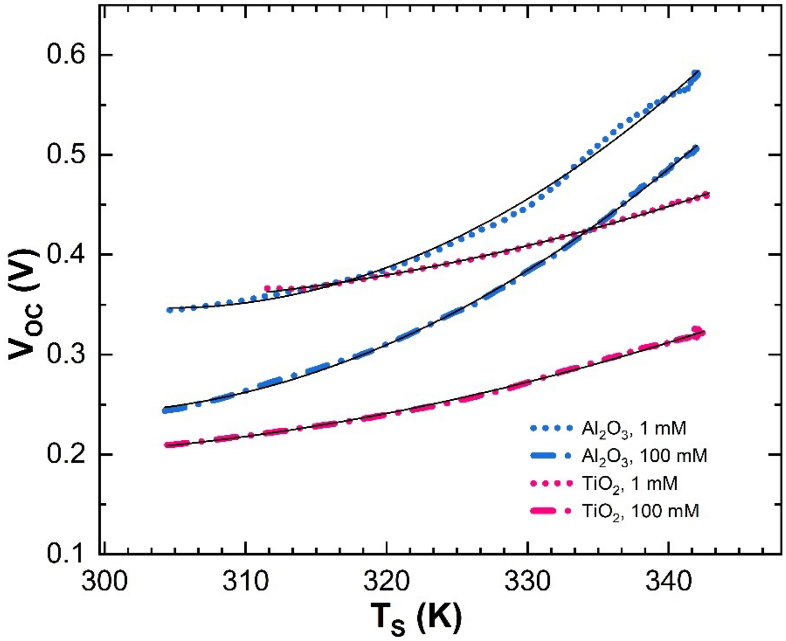

Figure S12: *Measured V_{OC} with temperature and the fit with the quadratic line. The fitting parameters is given in Table S1.*

Table S1: *Value of the fitting parameters obtained by using a quadratic fit as shown in Figure S12.*

Material (i)	*Conc. (j)*	*A_{ij}*	*B_{ij}*	*C_{ij}*
Al_2O_3	1 mM	1.7e-4	-0.01	0.517
	100 mM	1.4e-4	-0.007	0.331
TiO_2	1 mM	5.0e-5	-0.0023	0.376
	100 mM	4.0e-5	-0.001	0.2

S13: Derivation of the expression for $r_{3\Phi}$ for 1 mM and 100 mM concentrations

The expression for open circuit voltage as a function of temperature for the two dielectric shell materials Al_2O_3 (i=1), and TiO_2 (i=2), and concentrations of electrolyte 1 mM (j=1), and 100 mM (j=2) is given by:

$$V_{OC}^{ij}(T) = \left(v_f \overline{\sigma} r_{3\Phi}\right)^j + \Phi^{ij}\left(1 + \left(\frac{v_f r_{3\Phi}}{R_{sol}}\right)^j (R_{sol}C_{tr})^{ij}\right) \qquad (S12)$$

It is reasonable to assume, that the variables like $v_{f}, \overline{\sigma}, r_{3\Phi}$ does not change when the materials on the bottom SiNPs electrode changes, as long as the top electrode are the same. This allows us to determine the expression for $v_f r_{3\Phi}$as a function of temperature for different electrolyte concentrations.

The chemical potential difference, Φ, can be expressed as a linear function of temperature as follows, where Φ_0 is at ambient temperature T_0. The slope, k, is obtained from the linear part of the voltage-temperature curves. The value of Φ_0 is approximated as $V_{\Delta T}$, expressed by linear-temperature dependence (**S9**) as:

$$\Phi^{ij} = \Phi_0^{ij} + k^{ij}(T_S - T_0) \qquad (S13)$$

where, the slopes for different materials (TiO_2 or Al_2O_3) are obtained from the experimental fit of the linear part of the V_{oc}-time traces as:

$$k^{ij} = \begin{bmatrix} 0.0041 & 0.0045 \\ 0.0025 & 0.0027 \end{bmatrix} (VK^{-1}) \qquad (S14)$$

Thus, we obtained the functional dependence of $r_{3\Phi}$ with temperature for different concentrations using the measured open circuit voltage and our proposed model.

$$\left(\frac{v_f r_{3\Phi}}{R_{sol}}\right)^j = \frac{V_{OC}^{1j}(T) - V_{OC}^{2j}(T) - (\Phi^{1j} - \Phi^{2j})}{(R_{sol}C_{tr}\Phi)^{1j} - (R_{sol}C_{tr}\Phi)^{2j}} \qquad (S15)$$

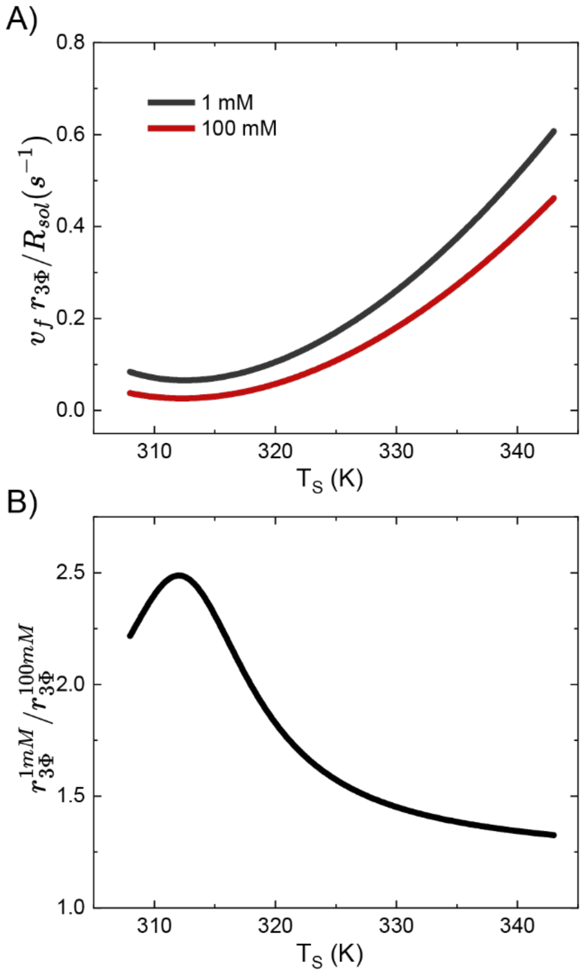

Figure S13: Estimating the functional form of 3-phase resistance. A) *Normalized by the solution resistance, which is a function of temperature.* ***B)*** *Ratio of 3-phase resistance as a function of temperature normalized by the ratio of electrolyte conductivities (*$^{conductivity\ of\ 100\ mM\ KCl}/_{conductivity\ of\ 1\ mM\ KCl} \approx 84$*) at an ambient temperature of 25 °C.*

S14: Derivation of differential capacitance for the Electrical Double Layer

$$\Phi = \frac{2k_B T_S}{e}\sinh^{-1}\left(\frac{\sigma(T_S)}{\sqrt{8000\epsilon_0\epsilon_r c_0 k_B T_S}}\right) + \frac{\sigma(T_S)}{C_{stern}} \qquad (S16)$$

The differential capacitance is given by:

$$C = \frac{\partial\sigma}{\partial\Phi}$$

$$\frac{1}{C_{DL}} = \frac{1}{C_d} + \frac{1}{C_{stern}} = \frac{\partial\Phi}{\partial\sigma} \qquad (S17)$$

$$\frac{\partial\Phi}{\partial\sigma} = \frac{2k_B T_S}{e}\frac{1}{\sqrt{8000\epsilon_0\epsilon_r c_0 k_B T_S + \sigma^2}} + \frac{1}{C_{stern}}$$

$$C_d = \frac{e}{2k_B T_S}\sqrt{8000\epsilon_0\epsilon_r c_0 k_B T_S + \sigma^2} \qquad (S18)$$

S15: Impedance measurement

1. Rationale for Selecting 1 kHz:

To determine the optimal frequency for real-time impedance monitoring, we initially conducted a full electrochemical impedance spectroscopy (EIS) scan over a frequency range of 10^6 to 10^2 Hz. Based on the typical nature of the EIS plots, the high frequency region is related to the charge kinetics, while the low frequency region is the diffusion-controlled region. Furthermore, we obtained the Bode plots from this scan, and we analyzed them to understand the system's characteristic impedance behavior. We noticed a transition for both the phase and amplitude plots around 1kHz. Based on this analysis, 1 kHz was selected as the monitoring frequency for time-resolved impedance measurements (commonly referred to as single-frequency impedance (SFI) monitoring). This frequency captures the relevant interfacial dynamics with sufficient signal stability.

2. Capacitance Estimation Method:

At 1 kHz, the impedance was measured by recording both the amplitude and the phase. From these data, the real (resistive) and imaginary (capacitive) components of the impedance were

extracted. The capacitance was estimated using the imaginary component of impedance (Z'') at the selected frequency, following the standard relation:

$$C = \frac{1}{2\pi f\ |Z''|} \qquad (S19)$$

f=1kHz is the measurement frequency. This approach enables dynamic tracking of capacitive behavior in response to changes in illumination or temperature.

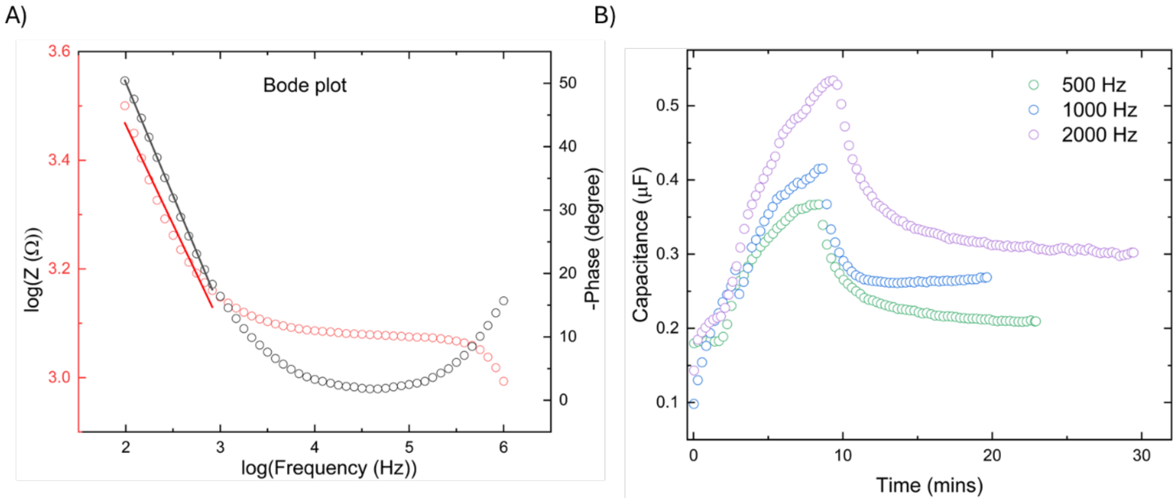

Figure S14: Electrochemical impedance spectroscopy and frequency-dependent capacitance analysis. A) *Bode plot obtained from electrochemical impedance spectroscopy (EIS) measurements, illustrating the frequency dependence of the impedance magnitude and phase of the system.* ***B)*** *Time-traces of the capacitance recorded at different frequencies during successive heating and cooling cycles, during which the temperature was continuously varied.*

S16: Open circuit voltage measured for different doping of the silicon core

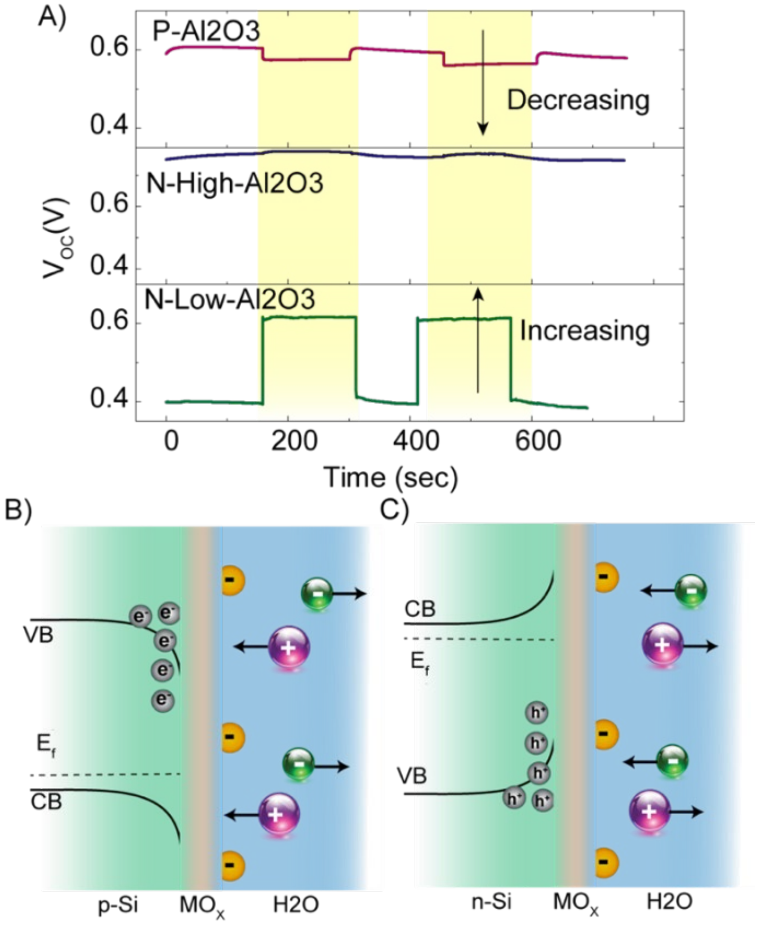

Figure S15: Effect of silicon core doping on the open-circuit voltage and interfacial band bending. ***A)*** *Time trace of the measured open circuit voltage at 1 mM KCl for 3 devices with different doping of the silicon core but with the same* Al_2O_3 *shell. The test was performed in ambient conditions and under 1 Sun illumination (shaded region).* ***B)*** *Band bending at p-silicon-oxide-electrolyte interface showing accumulation of electrons and movement of cations towards the interface.* ***C)*** *Band bending at n-silicon-oxide-electrolyte interface showing accumulation of holes and movement of cations away from the interface.*

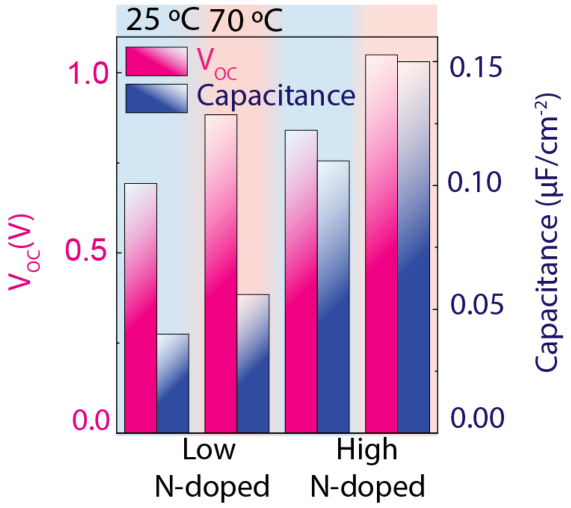

Figure S16: *Steady-state open circuit voltage (pink bars) and capacitance (blue bars) values for two devices with Al_2O_3 shell, but with different doping of silicon core (low N-doped: 1-20 Ω.cm and high N-doped: <0.005 Ω.cm) at 1 mM KCl. The blue and red shaded region is measured at a surface temperature of $T_S = T_{ambient}$ = 25°C and T_S = 70 °C, respectively.*

S17: Measuring photovoltage at different initial surface charges

The photovoltage at different temperatures was obtained by first subtracting the dark voltage baseline and then using $V_{ph} = V_{OC}^{light} - V_{OC}^{dark}$ to obtain the photovoltages.

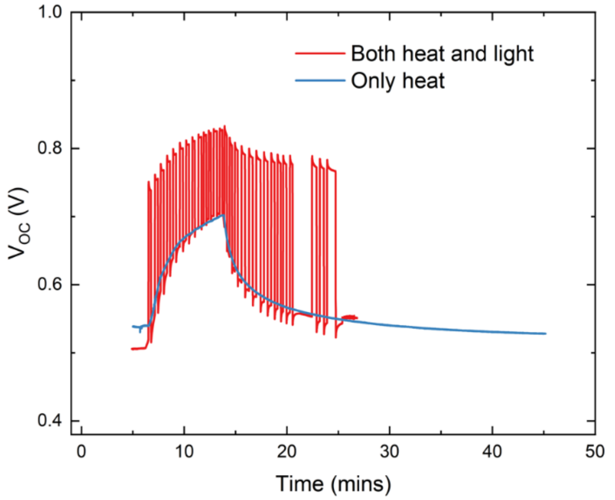

***Figure S17**: Measured V_{OC} during the heating and cooling cycle (depicted by the blue curve). Measured V_{OC} subjecting the system to the heating and cooling cycle with periodic activation and deactivation of solar light at an intensity of 100 mW/cm² (red curve). The experiments were performed at different absolute times after the sample was dried and replenished with fresh electrolyte.*

S18: Time-trace of Capacitance measurements during the heating and cooling phases for different electrolytes

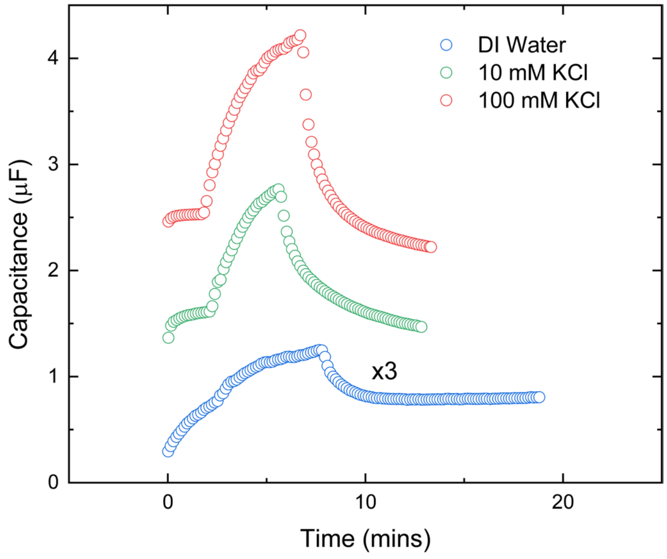

Figure S18: *Measured capacitance for different electrolytes under varying temperatures during the heating and cooling phases of the electrode using the heater placed beneath it. The value for the DI water case is multiplied by 3 for graphical visualization.*

Table S2: Fitting parameters for photovoltage-intensity lines for different wavelengths

Irradiation	Intercept Value	Intercept Std. Error	Slope Value	Slope Std. Error	Adj. R-Square
450 nm	0.12419	0.00107	0.05679	8.49642E-4	0.99799
515 nm	0.19430	6.80517E-4	0.07904	4.89904E-4	0.99965
600 nm	0.21553	0.00197	0.08133	0.00110	0.99836
630 nm	0.25127	4.06167E-4	0.09280	2.97459E-4	0.99991
660 nm	0.26404	7.73960E-4	0.09677	6.91200E-4	0.99954
730 nm	0.29055	0.00339	0.10140	0.00291	0.99264
850 nm	0.32750	0.00612	0.11257	0.00480	0.98389
950 nm	0.29022	3.74591E-4	0.10099	3.66316E-4	0.99988
Solar	0.24090	0.00169	0.09004	0.00369	0.98505

S19: Absorption spectra of the silicon electrode

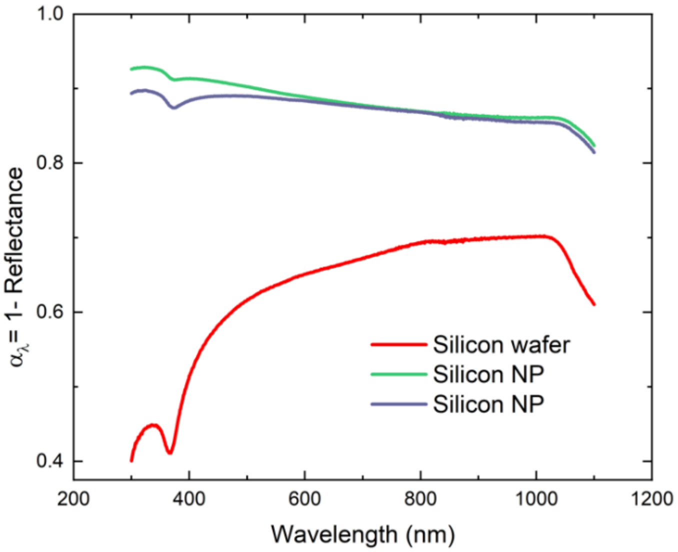

Figure S19: *Absorption measurement for the silicon wafer and the two silicon nanopillar array samples. Regarding the light intensity reaching the electrode surface, we have included absorption measurements for the silicon nanopillar sample. Additionally, we have provided the absorption measurement of the original silicon substrate as a reference.*

S20: Charge-regulation, Band-bending, and Photovoltage

I: Surface charge regulation:

Here, we conducted a thorough analysis of the liquid-solid interfacial surface charge and potential, with a specific emphasis on the silicon-electrolyte system featuring a few-nanometer layer of oxide (**Figure S20-I A-inset**). We will firmly establish the regulatory mechanisms of surface charge, which are influenced by variations in electrolyte properties and the corresponding chemical equilibria, as well as the significant effects of surface band-bending and the inherent properties of the silicon and oxide layer.

Once the electrical double layer (EDL) is formed, counter charges are attracted to the solid side, which, in the case of a metal electrode, are typically located just beneath the surface. However, for semiconductor materials, characterized by a significantly lower carrier density compared to metals, these counter charges can extend deep into the substrate, resulting in a pronounced space charge layer similar to that found in purely solid-state devices. In analyzing a solid-liquid system, we thus begin with a scrutiny of the surface band bending of silicon, which produces a distinct potential profile and a built-in electric field within the space charge region. This

potential profile is heavily influenced by key parameters, including the doping concentration of the silicon substrate, the density of surface states, and the bulk potential of the silicon relative to the bulk electrolyte. In the numerical calculation presented below, we have set the bulk potential of the electrolyte as zero and used the bulk potential of silicon (V_{Si}) as a free parameter. As these calculations are purely electrostatic in nature, we want to emphasize that the variation in V_{Si} can be understood analogously in terms of the generation of open-circuit voltage in the device at a given set of conditions. Consequently, these factors profoundly impact the electrical potential and the distribution of ionic species in the EDL, which plays a crucial role in determining the charge and potential at the surface. In the next part of this section, we will show how the generation of photovoltage is linked to band-bending and potential profiles across the interface.

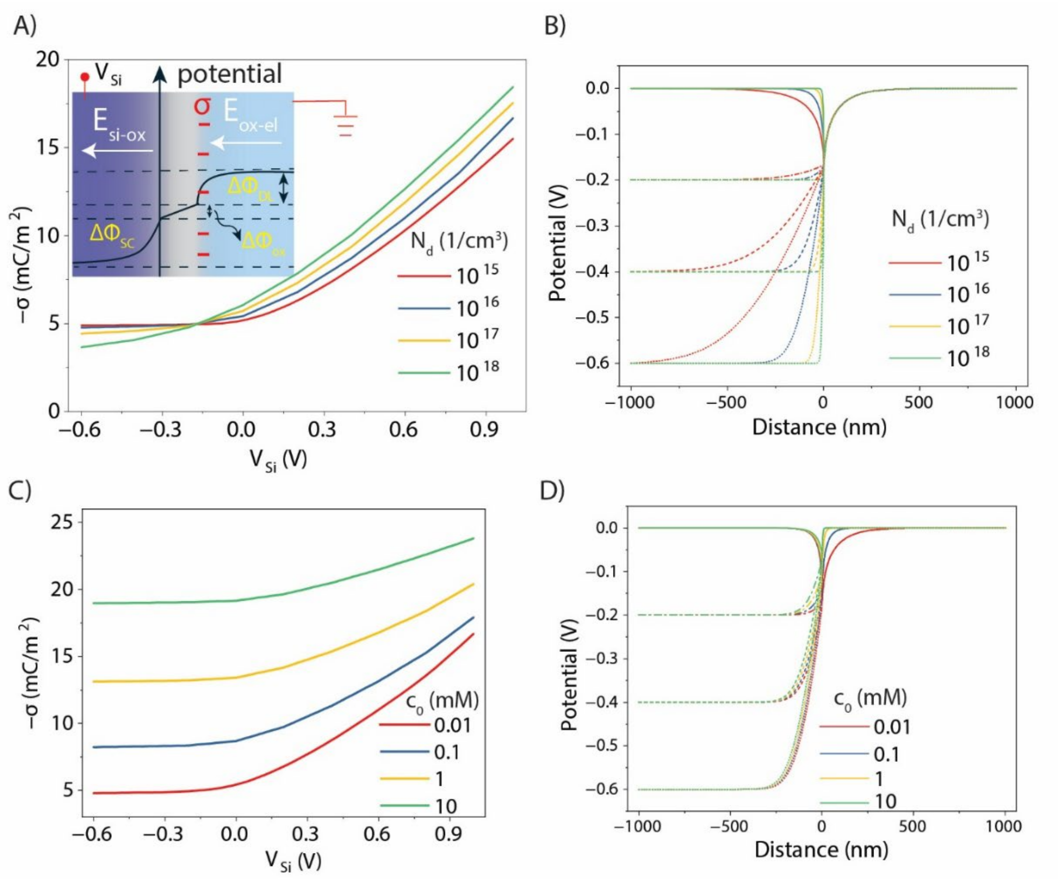

Figure 20-I: Influence of band bending at the silicon–oxide side on interfacial charge and electric field regulation. ***A)*** *Calculated surface charge at the solid–liquid interface for n-type silicon under different dopant concentrations (1/cm³). The inset illustrates how changes in band bending modify the interfacial potential profile, thereby shifting the chemical equilibrium on the electrolyte side.* ***B)*** *The potential profile across the semiconductor-oxide electrolyte system for various bulk potentials of silicon and dopant concentrations.* ***C)*** *Surface charge variation as a function of bulk potential of silicon for various electrolyte concentrations.* ***D)*** *Corresponding potential profile for various bulk potentials of silicon and electrolyte concentrations.*

II: Surface photovoltage:

The absorption of light above the semiconductor's band gap generates electron-hole pairs, which are effectively separated by the electric field induced by the initial band bending in the absence of light. This process significantly reduces band-bending, ultimately leading to a flatter response at high intensities. As a result, this phenomenon culminates in the generation of surface photovoltage. The maximum photovoltage is directly correlated with the initial band-bending observed in the dark, which is heavily influenced by the doping levels of silicon[7]. As illustrated in **Figure S20-II D**, as band-bending levels escalate from $2k_BT$ to $5k_BT$, the saturation value of photovoltage correspondingly increases. Furthermore, **Figure S20-II B** demonstrates the relationship between band bending and surface state density across various doping concentrations. It is evident that low-doped silicon experiences rapid saturation in band bending, resulting in a significantly larger band bending compared to high-doped silicon. Consequently, this leads to a greater photovoltage under illumination, clearly explaining why low-doped silicon achieves larger photovoltage values than its high-doped counterparts. *Note: For simplicity, the analysis presented here doesn't take into account the effect of the electrical double layer.*

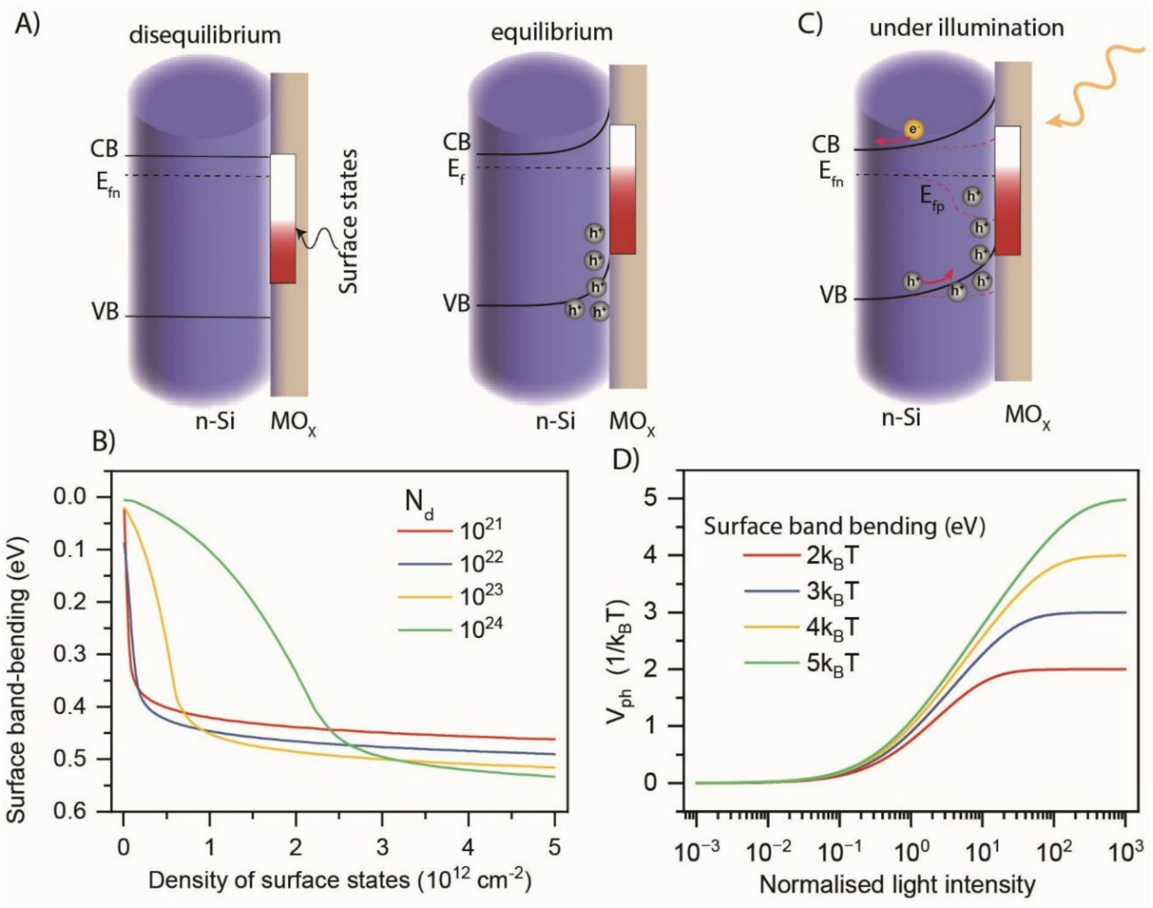

Figure S20-II: Band structure and photoresponse at the semiconductor-oxide interface. A) *Band diagrams before (left) and after (right) equilibration, showing how surface states fill to align the Fermi levels across the interface. The red–white gradient illustrates progressive state filling during equilibration.* ***B)*** *Calculated surface band bending for n-type silicon as a function of surface state density and dopant concentration. Band bending saturates once the Fermi level approaches the surface states.* ***C)*** *Under illumination, splitting of the quasi-Fermi levels for electrons and holes occurs, leading to band flattening. The separation between these quasi-Fermi levels corresponds to the measurable photovoltage.* ***D)*** *Calculated photovoltage as a function of light intensity for different initial band-bending conditions. The saturation value increases with the magnitude of dark band bending.*

S21: Equivalent circuit based on current and voltage sources. Derivation of the transient open circuit voltage.

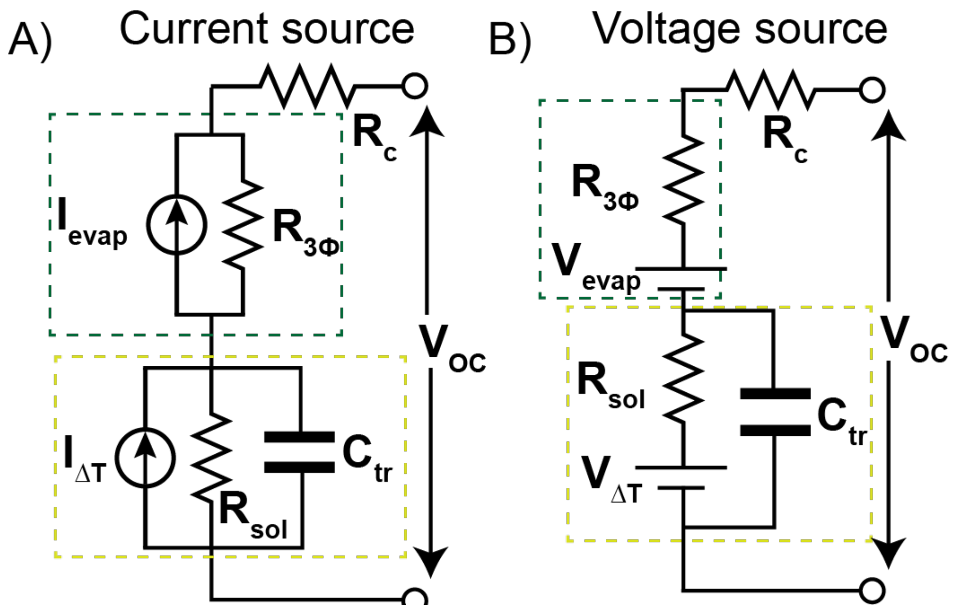

Figure S21: Equivalent electrical circuit for estimating open circuit voltage*. *A) *with a current source. **B)** with a voltage source.*

In the transient condition, the charge stored in the capacitor is governed by the following linear differential equation, obtained based on Kirchhoff's loop law.

$$\frac{dq_c}{dt} + \frac{q_c}{R_{sol}C_{tr}} = \frac{V_{\Delta T}}{R_{sol}} \qquad (S20)$$

$$q_c(t) = C_{tr}V_{\Delta T}e^{-\frac{t}{R_{sol}R_{ctr}}} \qquad (S21)$$

Thus, the open circuit voltage is given by

$$V_{OC}(t) = V_{evap} - V_{\Delta T}\left(1 - e^{-\frac{t}{R_{sol}R_{ctr}}}\right) \qquad (S22)$$

S22: Open circuit voltage measured with ethanol

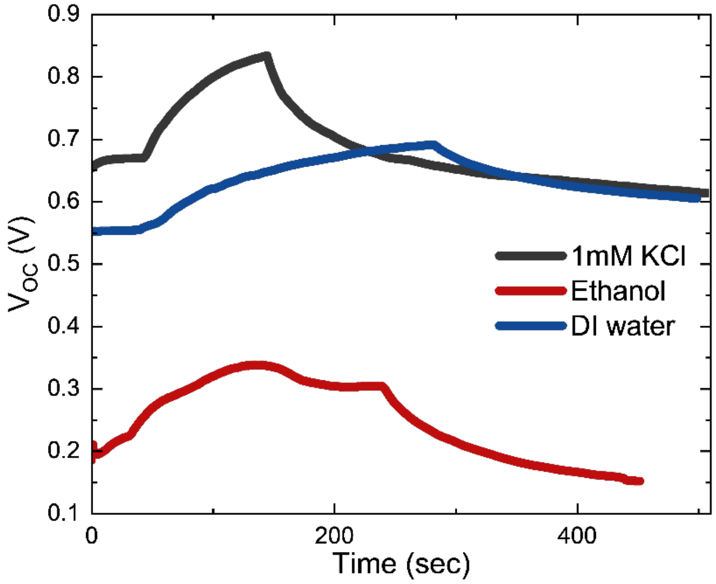

Figure S22: *Time trace of the open circuit voltage for a representative sample with different solvents. The time trace shows an initial case of ambient temperature and a heating phase, followed by a cooling phase. The voltage is significantly lower for ethanol compared to water. Furthermore, having ions in the solvent (1mM KCl cases) increases the open circuit voltage due to the higher chemical potential difference.*

In our system, the charge flux associated with evaporation-driven ion transport is governed by the relation (as shown in methods in the manuscript):

$$I_{Evap} = Wv_f(\bar{\sigma} + C_{tr}\Phi) \qquad (S23)$$

Polar solvent molecules play a crucial role in modulating I_{Evap}, as well as ionic species such as H^+, OH^-, K^+, and Cl^- , through $\bar{\sigma}$ and Φ respectively

To experimentally assess the solvent's role, we conducted comparative measurements using two different polar solvents—deionized water and ethanol—as well as 1mMKCl in Deionized water, under otherwise identical conditions. The results show a clear difference in open-circuit voltage (V_{OC}):

- For DI water: V_{OC} = 0.552 V (25 °C) and 0.690 V (65 °C)
- For ethanol: V_{OC} = 0.195 V (25 °C) and 0.336 V (65 °C)
- For 1 mM KCl in DI water: V_{OC} = 0.652 V (25 °C) and 0.840 V (65 °C)

S23: Quantification of Transfer Capacitance

Determining the transfer capacitance involves a complex analytical expression due to the intricate geometry of the various interfacial charge storage layers[6]. However, we can confidently navigate this complexity.

As illustrated in **Figure S23**, the total transfer capacitance is clearly decomposable into contributions from both the bottom and top regions, represented by C1 and C2, respectively.

We begin by identifying the interfacial region at the bottom electrode. The yellow rectangle in **Figure S23** highlights both flat and cylindrical regions. We can accurately estimate the capacitance using the established formulations for flat plate and cylindrical capacitors, as detailed below.

$$C_1 = \frac{2\pi\varepsilon_0\varepsilon_r N L_{np}}{log\left(\frac{d_{np}+2\lambda_D}{d_{np}}\right)} + \frac{\varepsilon_0\varepsilon_r}{\lambda_D} \qquad (S24)$$

Where N represents the density of Nanopillars per unit area of the sample. L_{np} and d_{np} are the length and diameter of the nanopillars. $\varepsilon_0\varepsilon_r$ respectively, of the dielectric permittivity of the electrolyte. By considering a hexagonal arrangement of pillars with pitch, p, and diameter d_{np}, we can estimate the number density as:

$$N = \frac{2}{\sqrt{3}p^2}$$

Furthermore, the Debye length is expressed as:

$$\lambda_D = \sqrt{\frac{\varepsilon_0\varepsilon_r k_B T}{2e^2 C_b}} \qquad (S25)$$

Where C_b is the bulk ionic concentration of the electrolyte, e is the electronic charge, k_B is the Boltzmann constant, and T is the temperature in Kelvin.

We begin by identifying the interfacial region in the upper section, which consists of three distinct components, as illustrated in **Figure S23:**

1. The flat portion of the top electrode surface

2. The cylindrical segment of the top electrode surface

3. Two segments representing the spherical region at the liquid-vapor meniscus interface

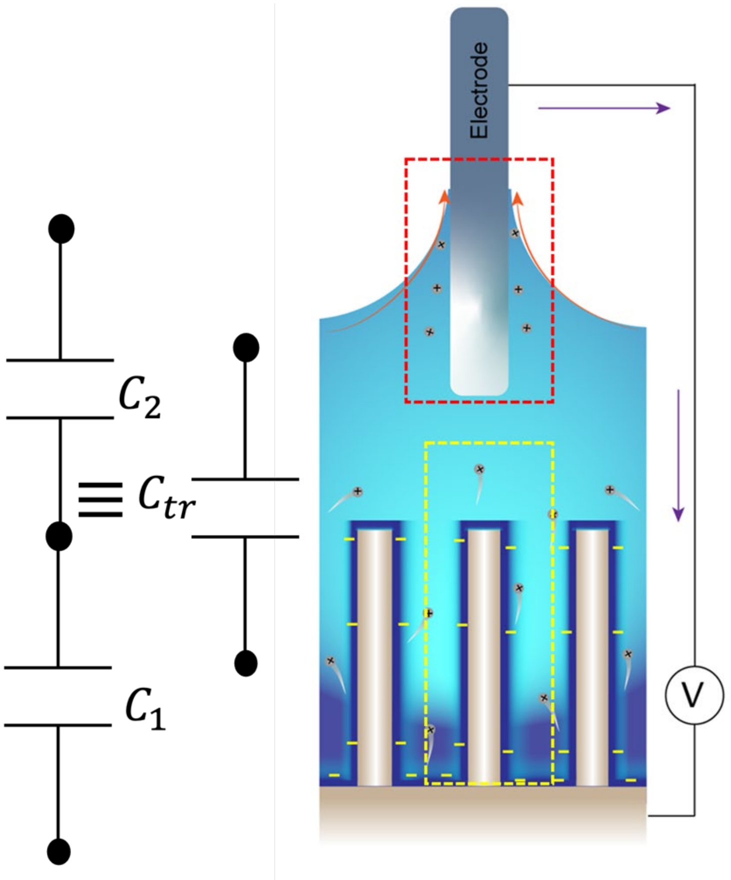

Figure S23: *Transfer capacitance estimation by identifying different interfacial regions of the bottom and top segments of the system.*

By considering the contributions of each component, we can derive an expression for capacitance that encompasses the capacitance formulations for the flat plate, cylindrical, and spherical shells with radius a. This comprehensive approach ensures that we capture the full complexity of the system and its impact on performance.

$$C_2 = \frac{1}{\pi a^2}\left[\frac{2\pi\varepsilon_0\varepsilon_r L_e}{log\left(\frac{d_e + 2\lambda_D}{d_e}\right)} + \frac{\varepsilon_0\varepsilon_r}{\lambda_D}\frac{\pi d_e^2}{4} + 2\pi\varepsilon_0\varepsilon_r\frac{a(a+\lambda_d)}{\{(a+\lambda_d)-a\}}\right] \quad (S26)$$

As a >> λ_D we can simplify it to get:

$$C_2 = \frac{1}{\pi a^2}\left[\frac{2\pi\varepsilon_0\varepsilon_r L_e}{log\left(\frac{d_e + 2\lambda_D}{d_e}\right)} + \frac{\varepsilon_0\varepsilon_r}{\lambda_D}\frac{\pi d_e^2}{4} + 2\pi\varepsilon_0\varepsilon_r\frac{a^2}{\lambda_D}\right] \quad (S27)$$

Further rearrangement led to:

$$C_2 = \frac{2\pi\varepsilon_0\varepsilon_r L_e}{log\left(\frac{d_e + 2\lambda_D}{d_e}\right)} \frac{1}{\pi a^2} + \frac{\varepsilon_0\varepsilon_r}{\lambda_D}\frac{d_e^2}{4a^2} + \frac{2\varepsilon_0\varepsilon_r}{\lambda_D} \qquad (S28)$$

By considering that $d_e^2 \ll a^2$ as $\pi a^2 = 1 cm^2$, and $d_e = 0.1$ cm, we can further simplify the expression by neglecting the second term in the above expression

$$C_2 = \frac{2\pi\varepsilon_0\varepsilon_r L_e}{log\left(\frac{d_e + 2\lambda_D}{d_e}\right)} \frac{1}{\pi a^2} + \frac{2\varepsilon_0\varepsilon_r}{\lambda_D} \qquad (S29)$$

Finally, as noted above, $\pi a^2 = 1\ cm^2$, we have the final expression for the capacitance C_2 as:

$$C_2 = \frac{2\pi\varepsilon_0\varepsilon_r L_e}{log\left(\frac{d_e + 2\lambda_D}{d_e}\right)} + \frac{2\varepsilon_0\varepsilon_r}{\lambda_D} \qquad (S30)$$

We repeat the final expression relevant for estimating the transfer capacitance below:

$$C_1 = \frac{2\pi\varepsilon_0\varepsilon_r N L_{np}}{log\left(\frac{d_{np} + 2\lambda_D}{d_{np}}\right)} + \frac{\varepsilon_0\varepsilon_r}{\lambda_D}$$

$$C_2 = \frac{2\pi\varepsilon_0\varepsilon_r L_e}{log\left(\frac{d_e + 2\lambda_D}{d_e}\right)} + \frac{2\varepsilon_0\varepsilon_r}{\lambda_D}$$

Using the Taylor expansion of the natural logarithm function, we can write:

$$\log(1 + x) = x - \frac{x^2}{2} + \frac{x^3}{3} - \frac{x^4}{4} \cdots$$

Neglecting higher-order terms as $\frac{\lambda_D}{d_{np}} \ll 1$, (Here we keep the terms up to third order, as at very low concentration, Debye length can be ~100 nm, which is similar to the diameter of the nanopillars, ~300-400 nm. We can simplify the expression for C_1 as:

$$C_1 = \frac{\varepsilon_0\varepsilon_r}{\lambda_D}\left[1 + \frac{\pi N d_{np} L_{np}}{1 - \frac{2\lambda_D}{d_{np}} + \frac{4}{3}\frac{\lambda_D^2}{d_{np}^2}}\right] = \frac{\varepsilon_0\varepsilon_r}{\lambda_D}(1 + K_1) \qquad (S31)$$

K_1 depends on geometrical parameters of the nanopillar array and the Debye length as:

$$where, \qquad K_1 = \frac{\pi N d_{np} L_{np}}{1 - \frac{2\lambda_D}{d_{np}} + \frac{4}{3}\frac{\lambda_D^2}{d_{np}^2}}$$

Neglecting non-linear terms as $\frac{\lambda_D}{d_{np}} \ll 1$, we can simplify the expression for C_2 as:

$$C_2 = \frac{\varepsilon_0 \varepsilon_r}{\lambda_D}\left[2 + \frac{\pi d_e L_e}{1(cm^2)}\right] = \frac{\varepsilon_0 \varepsilon_r}{\lambda_D}(2 + K_2) \qquad (S32)$$

K_2 depends on geometrical parameters of the electrodes, its meniscus shape, and the Debye length as:

$$where, \qquad K_2 = \frac{\pi d_e L_e}{\pi a^2}$$

We can obtain the expression for transfer capacitance that depends on the geometrical parameters of the bottom nanostructures, as well as the size of the top electrode and the features of the related meniscus regions.

$$C_{tr} = \frac{\varepsilon_0 \varepsilon_r}{\lambda_D}\left[\frac{(1 + K_1)(2 + K_2)}{3 + K_1 + K_2}\right] \qquad (S33)$$

S24: Evaporation rate dependence on temperature

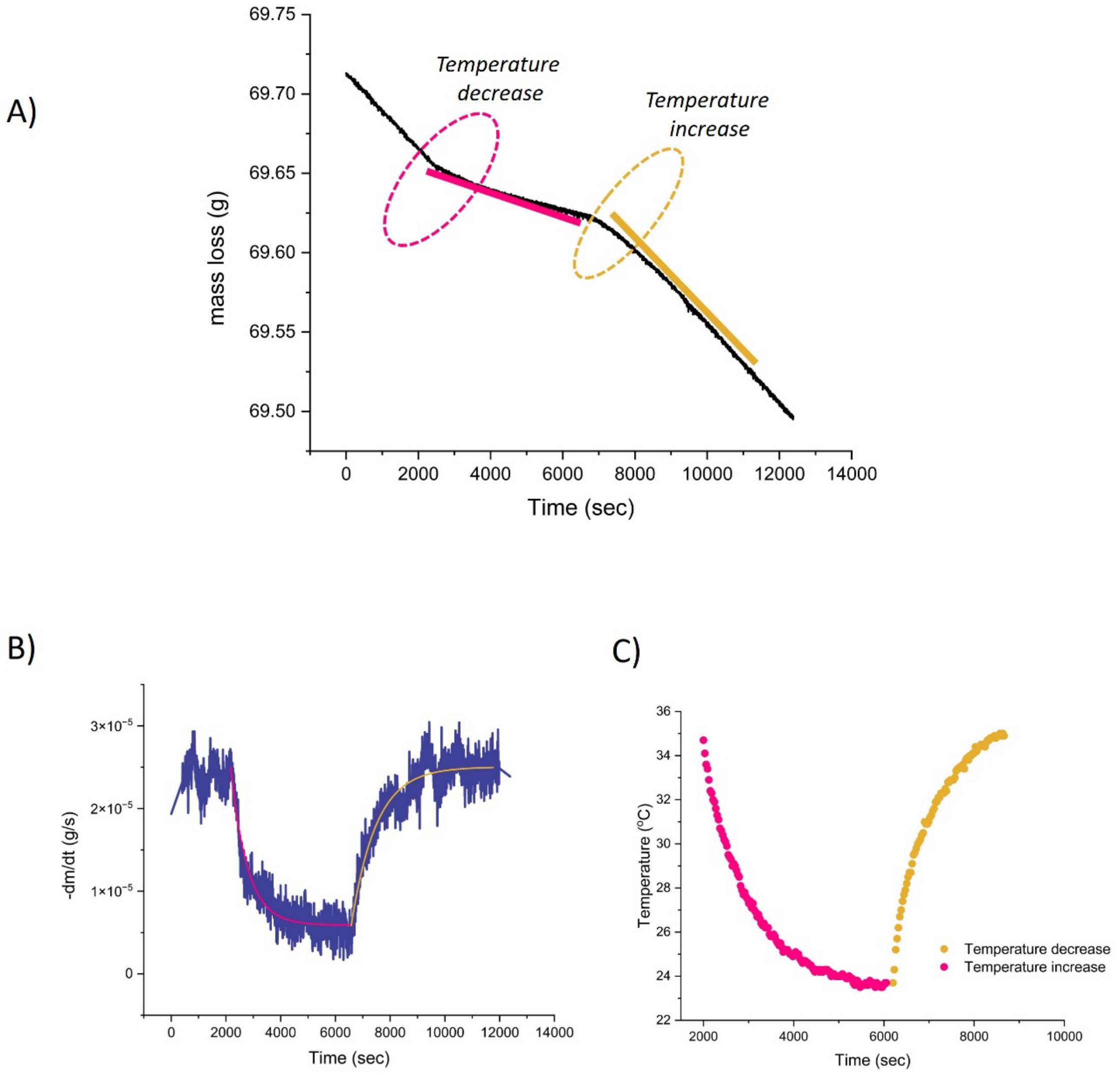

Figure S24: Evaporation rate measurement. A) *Mass loss from the system due to evaporation. Before the 2000s, the system was at ~35°C. After ~2000s (highlighted in pink), the system undergoes natural cooling to room temperature. Finally, the system undergoes a temperature increase (highlighted in yellow). See SI 11 for details on temperature measurements and the explanation for the different slopes during the temperature increase and decrease.* ***B)*** *The derivative of mass change. The pink and yellow lines represent exponential fits during the cooling and heating cycles, respectively.* ***C)*** *The corresponding temperature during cooling(pink) and heating (yellow).*

S25: Power-Voltage curve

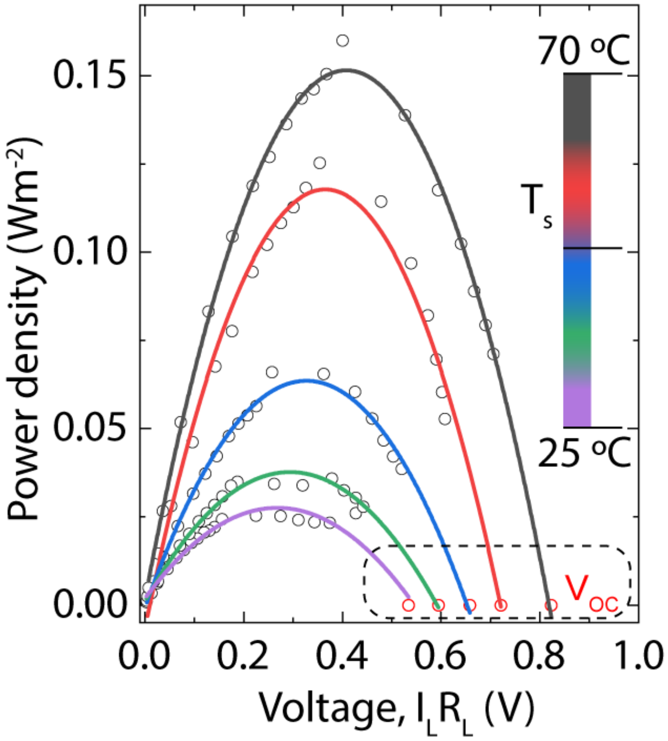

Figure S25: *Power-Voltage curve of the same device as reported in the manuscript* ***Figure 6c*** *for the same temperature conditions.*

S26: Obtaining the parameters from the power curves.

$$P(R_L) = \frac{\left[I_{\Delta T} R_{Sol}(1 + v_f r_{3\Phi} C_{tr}) + v_f \bar{\sigma} r_{3\Phi} + V_{ph}\right]^2 R_L}{\left[R_{Si} + R_{Sol}(1 + v_f r_{3\Phi} C_{tr}) + r_{3\Phi}/W + R_c + R_L\right]^2} = \frac{A(T, I, c_0) R_L}{[B(T, I, c_0) + R_L]^2} \quad (S34)$$

The values of the parameters A and B obtained are shown below:

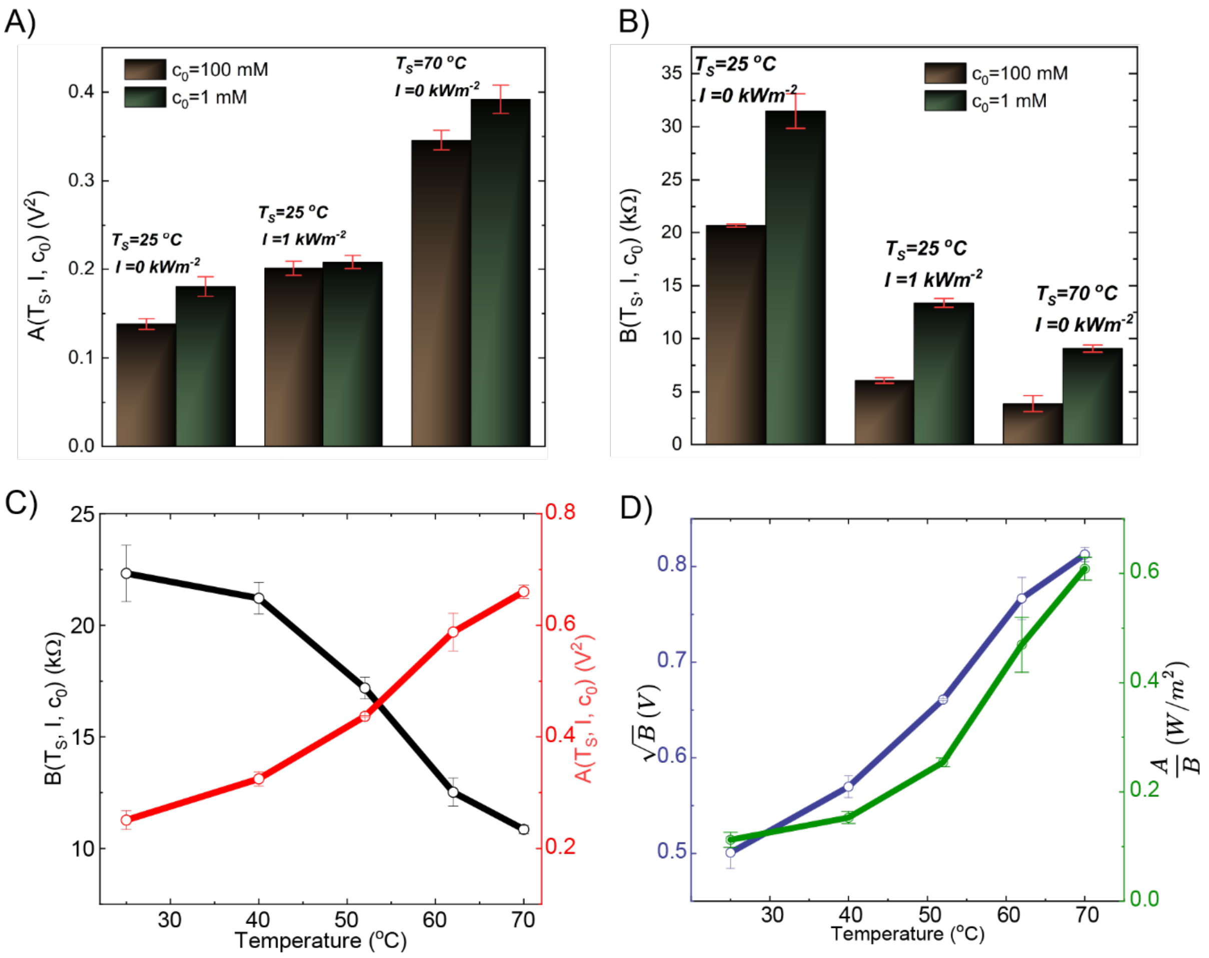

Figure S26: Determining the value of the parameters A and B under different conditions. A) *Values of A.* ***B)*** *values of B.* ***C)*** *Values of A (red curve) and B (black curve) at 1 mM concentration as a function of temperature when there is no incident light.* ***D)*** *Values of A and B in a different functional form, which scales as voltage (blue curve) and power (green curves).*

S27: Load line analysis for power estimation

In load line analysis, we systematically vary the external load resistance across a wide range and estimate the corresponding output voltage and current. This enables us to obtain the power output at a given loading condition. Additionally, we present the measured current–voltage (I–V) characteristics at different temperatures to highlight the influence of thermal conditions on device performance and load matching. These analyses collectively provide a comprehensive view of the electrical performance of our device under realistic operating conditions.

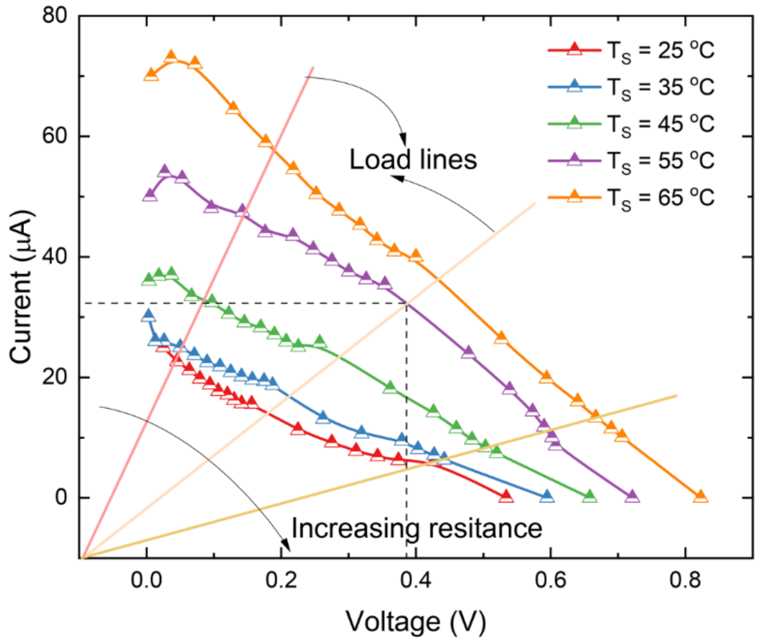

***Figure S27**: **Load line analysis of the device.** The current-voltage curves were measured at temperatures between 25 °C and 65 °C. The three straight lines passing through the origin are load lines with different resistances. The intersection of a specific load line with the current-voltage characteristic obtains the operating point. As a representative example, the intersection point of the purple curve with the two perpendicular dashed lines is shown.*

S28: calibration using a thermocouple

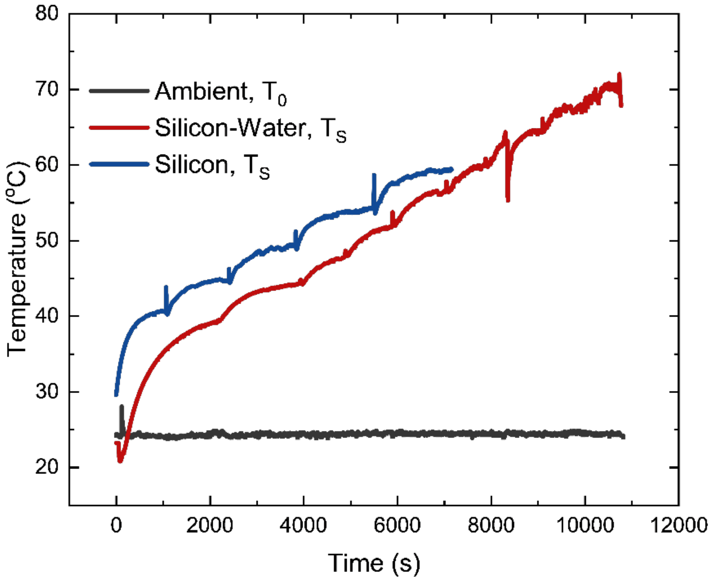

Figure S28*: Temperature of silicon substrate measured in the HV cell. The black curve shows the ambient temperature. The red and blue curves show the temperature of the silicon substrate with wetted and unwetted conditions of the experiments. The temperature is gradually increased by changing the voltage supplied to the heater until a maximum temperature of 70°C is reached. The slight temperature difference between the blue and red lines is due to the higher heat loss pathways in the wetted condition.*

S29: Repeatability test at very high concentrations of KCl

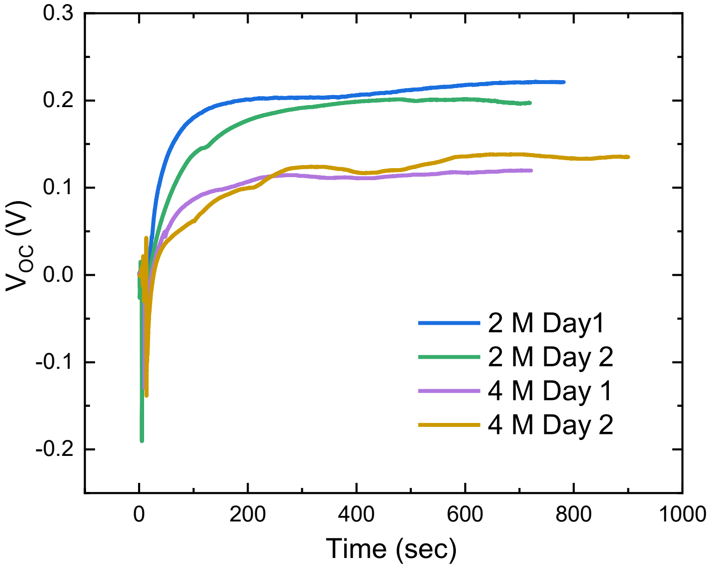

Figure S29: Repeatability test at high concentrations*. Time evolution of open circuit voltage from a particular sample at 2M and 4M KCl concentrations performed on two different days*[2]

S30: COMSOL model

We developed a 3D numerical model (COMSOL®) to solve the Nernst-Planck-Poisson equation to determine the equilibrium distribution of ions and resulting electrostatic potentials. For modeling, we considered different modes of ion transport, using the Nernst-Planck equation for dilute species and the Poisson-Boltzmann equation for the equilibrium distribution of ions. To perform these calculations, however, we must first identify an equivalent simplified geometry using the approach described in our prior work[2]. The cross-section of the simulated axis-symmetric geometry is shown below. The mesh elements are shown, with a boundary element mesh near the liquid-solid interface.

Governing equations:

$$\nabla \cdot J_i + U \cdot \nabla c_i = 0 \qquad (S35)$$

$$J_i = -D\nabla c_i - z_i \mu_i^m F c_i \nabla\Phi \qquad (S36)$$

$$\nabla^2\Phi = -\frac{1}{\epsilon_0\epsilon_r}\sum_i F z_i c_i exp\left(-\frac{e z_i \Phi}{k_B T_S}\right) \qquad (S37)$$

We used surface charge density as the boundary condition, which is not a fixed value but is instead governed by the above equilibrium reaction[2,8,9]. This results in variable surface charge density that depends on the surface potential at the stern plane, given by the following equation:

$$\sigma = \frac{-e\Gamma}{1 + \frac{[H^+]_S}{K_a}} \ , and$$

$$[H^+]_S = [H^+]_{bulk}\, exp\left(-\frac{e\Phi_S}{k_B T_S}\right) \qquad (S38)$$

The material's chemical characteristics Δ_H is the enthalpy of dissociation of the surface groups. Then, we swept Δ_H, and obtained voltage-temperature lines for various conditions. This enabled us to obtain the open-circuit voltage (surface charge) as a function of temperature for multiple conditions, such as varying electrolyte concentration, pH, and K_{a0}. Finally, we constructed the correlation between the slopes of voltage-temperature lines and their value Δ_H.

$$K_a = K_{a0}\ \exp\left[-\frac{\Delta_H}{R}\left(\frac{1}{T} - \frac{1}{T_0}\right)\right] \qquad (S39)$$

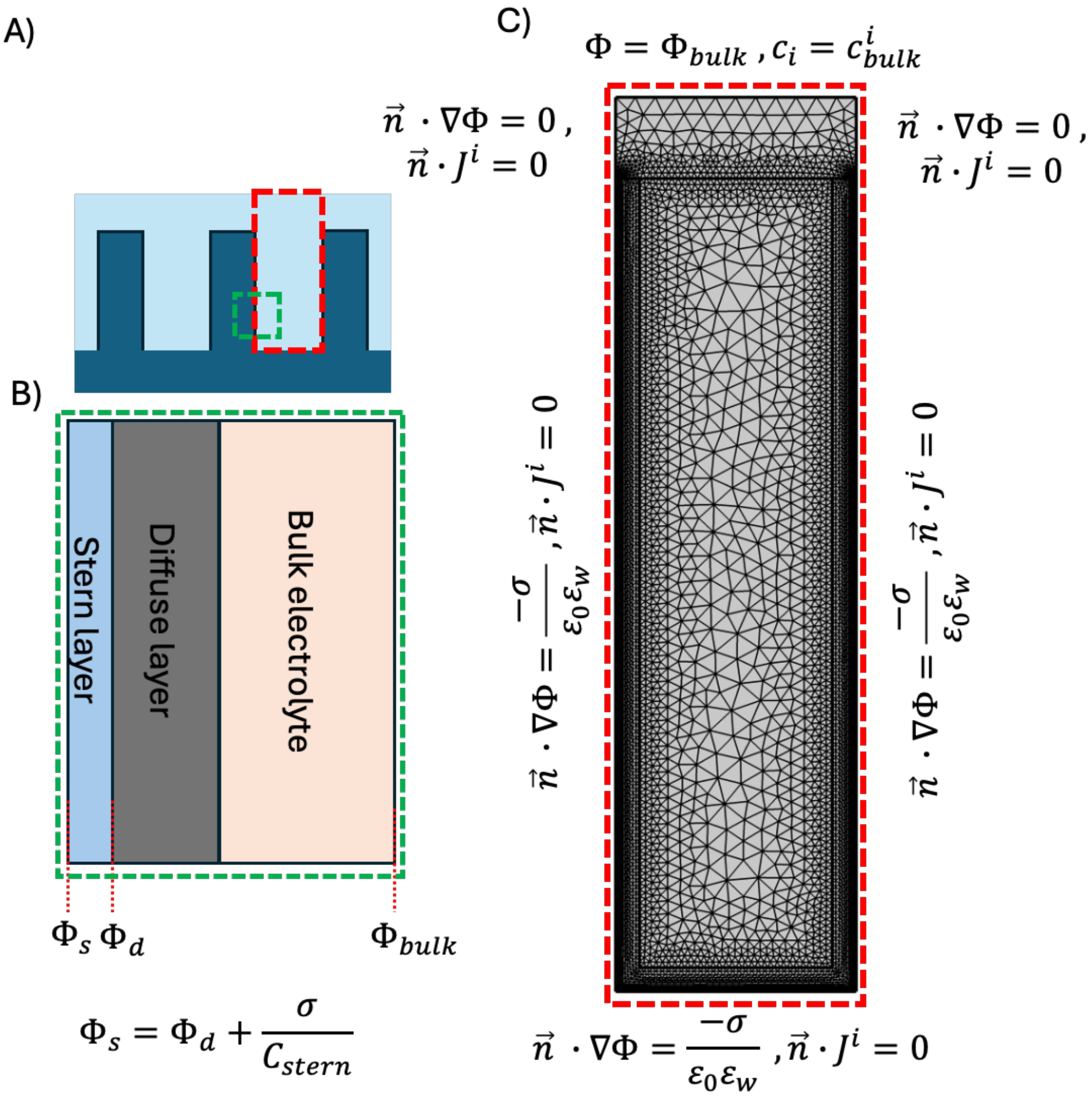

Figure S30: Numerical model geometry and boundary conditions. ***A)*** *Cross-section of the device's nanostructure with the region where the governing equations are solved (enclosed in red rectangle).* ***B)*** *Zoom-in view of the liquid-solid interface (shown in a green rectangle) with the potential at the stern plane and diffuse layer. The equation gives the relation between the potential at the stern and diffuse layer connected by the surface charge density and stern layer capacitance.* ***C)*** *Cross-section the simplified axis-symmetric geometry used in the numerical simulation after building the mesh. The boundary conditions for the electrical potential and ion flux are shown at different boundaries.*

Table S3: Value of the parameters used in the simulation.

Variable name	Values and units	Description
**T_o**	298.1 K	Ambient temperature
**t_{stern}**	0.5 nm	Stern layer thickness
**pH_b**	7	pH of bulk electrolyte
**V_{therm}**	$k_B T/q$	Thermal voltage
**W_{Ag}**	4.6 V	Work function of Ag
**E_{eq}**	0.2 V	Equilibrium potential of the reference electrode
**D_A**	1e-9 m^2/s	Diffusion coefficient, cation
**D_X**	1e-9 m^2/s	Diffusion coefficient, anion
**D_H**	36.3e-4[$cm^2/(Vs)$]*V_{therm}	Diffusion coefficient, H+
**D_{OH}**	20.5e-4[$cm^2/(Vs)$]*V_{therm}	Diffusion coefficient, OH-
**C_o**	0.001 M	Bulk electrolyte concentration
**cH_{bulk}**	10^{-pHb} M	Bulk H+ concentration
**cOH_{bulk}**	10^{-14}[M^2]/cH_{bulk}	Bulk OH- concentration
**cA_{bulk}**	c0+cOH_{bulk}	Bulk cation concentration
**cX_{bulk}**	c0+cH_{bulk}	Bulk anion concentration
**z_A**	1	Cation charge

Variable name	Values and units	Description
$\mathbf{z_X}$	-1	Anion charge
$\mathbf{I_{bulk}}$	0.5*($z_A{}^2$*cA_{bulk}+$z_X{}^2$*cX_{bulk} +cOH_{bulk}+cH_{bulk})	Bulk ionic strength
$\boldsymbol{\varepsilon_W}$	78.5	Relative permittivity of water
**F**	96485 C/mol	Faraday's constant
**R**	8.3145 J/mol.K	Univeral gas constant
$\boldsymbol{\lambda_D}$	$\sqrt{\frac{\varepsilon_0 \varepsilon_W V_{therm}}{2F I_{bulk}}}$	Debye length
$\boldsymbol{\varepsilon_{st}}$	12	Relative permittivity of Stern layer
$\mathbf{C_{st}}$	$\varepsilon_0 \varepsilon_W / t_s$	Stern layer capacitance
$\mathbf{K_{ao}}$	10^{-pKao} M	Equilibrium constant
**Γ**	8 x 10^{14} 1/cm^2	Oxide surface binding site density
$\boldsymbol{\Phi_{bulk}}$	-W_{Ag}-E_{eq}	Bulk electrical potential
$\mathbf{t_w}$	0.1 µm	Thickness of water layer
$\mathbf{K_a}$	$K_{a0}\ \exp\left[-\frac{\Delta_H}{R}\left(\frac{1}{T}-\frac{1}{T_0}\right)\right]$	Equilibrium constant at temperature *T*
$\boldsymbol{\Delta_H}$	50 kJ/mol	Enthalpy of dissociation
$\mathbf{pK_{ao}}$	6	Equilibrium constant at T_o

References

1. Wendisch, F. J., Rey, M., Vogel, N. & Bourret, G. R. Large-Scale Synthesis of Highly Uniform Silicon Nanowire Arrays Using Metal-Assisted Chemical Etching. *Chem. Mater.* **32**, 9425–9434 (2020).
2. Anwar, T. & Tagliabue, G. Salinity-dependent interfacial phenomena toward hydrovoltaic device optimization. *Device* **2**, 100287 (2024).
3. Thangamuthu, M., Santschi, C. & Martin, O. J. F. Reliable Langmuir Blodgett colloidal masks for large area nanostructure realization. *Thin Solid Films* **709**, 138195 (2020).
4. Droplet evaporation on porous fabric materials | Scientific Reports. https://www.nature.com/articles/s41598-022-04877-w.
5. Sposito, G. On the surface complexation model of the oxide-aqueous solution interface. *Journal of Colloid and Interface Science* **91**, 329–340 (1983).
6. Artemov, V. *et al.* The Three-Phase Contact Potential Difference Modulates the Water Surface Charge. *J. Phys. Chem. Lett.* **14**, 4796–4802 (2023).
7. Mönch, W. *Semiconductor Surfaces and Interfaces*. (Springer, Berlin ; New York, 2001).
8. Behrens, S. H. & Grier, D. G. The charge of glass and silica surfaces. *The Journal of Chemical Physics* **115**, 6716–6721 (2001).
9. Stein, D., Kruithof, M. & Dekker, C. Surface-Charge-Governed Ion Transport in Nanofluidic Channels. *Phys. Rev. Lett.* **93**, 035901 (2004).
